# Political economy analysis of health: a scoping review of concepts, definitions, frameworks, outcomes, and applications

**DOI:** 10.1093/heapol/czaf096

**Published:** 2026-06-29

**Authors:** Vineetha Karuveettil, Chandrashekar Janakiram, Shewta Ramesh, Aparna Ramachandran, Manu Mathur, Balagopal Varma, Heidi Green, Denny John

**Affiliations:** Department of Public Health Dentistry, Amrita School of Dentistry, Amrita Vishwa Vidyapeetham, Kochi 682041, India; JBI Amrita Centre for Evidence Synthesis and Implementation, Kochi 682041, India; Department of Public Health Dentistry, Amrita School of Dentistry, Amrita Vishwa Vidyapeetham, Kochi 682041, India; JBI Amrita Centre for Evidence Synthesis and Implementation, Kochi 682041, India; Department of Public Health Dentistry, Amrita School of Dentistry, Amrita Vishwa Vidyapeetham, Kochi 682041, India; Department of Public Health Dentistry, Amrita School of Dentistry, Amrita Vishwa Vidyapeetham, Kochi 682041, India; Institute of Dentistry, Queen Mary University of London, Turner Street, London, E1 2AD, United Kingdom; Department of Paediatric and Preventive Dentistry, Amrita School of Dentistry, Amrita Vishwa Vidyapeetham, Kochi 682041, India; Australian Centre for Health Engagement, Evidence and Values, School of Health and Society, University of Wollongong, Northfields Avenue, NSW 2500, Australia; The Centre for Transformative Nursing, Midwifery, and Health Research: A JBI Centre of Excellence, University of Newcastle, NSW 2308, Australia; Faculty of Life and Allied Health Sciences, MS Ramaiah University of Applied Sciences, Bengaluru 560054, India; Evidence Synthesis and Implementation for Indigenous Health: A JBI Affiliate Centre, Kolkata 700107, India

**Keywords:** health economics, health policy, health system, political economy, political science

## Abstract

This review examined 28 studies to understand how political economy analysis (PEA) is conceptualized and applied in health. Definitions of political economy varied, with only 11 studies offering explicit definitions. Most commonly, political economy was framed as the study of power, interests, institutions, and ideas shaping health policy processes and outcomes. Applications ranged from analysing structural determinants of health to understanding stakeholder influence in health reforms. Across studies, 31 distinct frameworks and theories were used. Frequently employed models included Campos and Reich’s Political Economy of Health Financing Reform Framework, Harris’s Applied PEA, and the DFID and World Bank ‘How-to’ notes. Theoretical underpinnings were drawn from economics, political science, and sociology—such as historical institutionalism, stakeholder theory, and discursive institutionalism—highlighting the interdisciplinary nature of PEA. Health issues analysed through a political economy lens primarily included health financing, governance, human resources for health, and service delivery. PEA was used to explore challenges such as policy reform feasibility, institutional capacity, health workforce equity, and donor dependency. The rationale for applying PEA included uncovering the influence of actors, navigating complex political contexts, and enhancing policy implementation. Overall, PEA in health is marked by conceptual diversity and methodological pluralism. Its growing application reflects the need to understand the interplay of politics, institutions, and economics in addressing systemic health challenges.

Key messages

- The political economy of health is variably defined across studies, but it broadly emphasizes power relations, institutional dynamics, and historical legacies that shape health policies, resource distribution, and governance structures.
- A diverse array of over thirty-one frameworks and theories—ranging from applied, stepwise models to complex theories in political science—has been employed to conduct political economy analyses of health.
- Political economy analysis has been applied across a broad spectrum of health system challenges, including human resources for health, governance, financing, service delivery, and information systems, particularly in complex or transitional contexts such as post-conflict settings or health reforms.
- Political economy analysis helps uncover how power, institutions, and socio-political dynamics shape health policies and systems, guiding more equitable and politically informed interventions.

## Introduction

Health is widely recognized as a fundamental human right, yet despite advances in medical technology and health care, stark disparities in health outcomes persist both within and between countries (Benatar 1998, Marmot et al. 2008, World Health Organisation 2020). Understanding these inequalities requires moving beyond biomedical models to examine the political and economic structures that shape health systems, individual experiences, and population outcomes. This interdisciplinary perspective is encapsulated in the political economy of health (PEH), which explores how economic doctrines, political institutions, and power relations interact to influence health and healthcare delivery (Nannini et al. 2021 ).

Political economy (PE) is often defined broadly as the study of interactions between political and economic systems, examining how political decisions influence economic outcomes (Drazen 2002). However, this definition risks oversimplification. A more nuanced understanding, following Maier’s foundational work, recognizes PE as an approach that interrogates economic doctrines to reveal their underlying sociological and political premises. In this view, economic ideas and behaviours are not merely analytical frameworks but are themselves social constructs and actions requiring explanation (Maier 1987). This critical stance on economic beliefs and policies is essential to grasp the influence of political economy on health fully.

Political economy analysis (PEA) operationalizes this approach by offering a methodology to identify and explain the political, economic, and social factors that shape policy processes and reforms. It offers a dual focus: reflecting on previous policy decisions and proposing forward-thinking strategies for current issues, providing opportunities to improve outcomes across various stages of the policy cycle (Heenan et al. 2022). Importantly, PEA is not confined to analysing policy formulation or reform design, but also extends to the implementation of policies, guidelines, and regulations. Implementation processes often expose the ‘real politics of reform’, where incentives, resources, and local power relations determine how policies are enacted in practice and why outcomes diverge across contexts (Corduneanu-Huci et al. 2012, Booth 2016). While institutions provide the structural rules of the game, PEA also draws attention to the role of actors—whether individuals, coalitions, or organised groups—whose choices, motivations, and incentives shape how institutions function in practice. Institutions are not static; they are created, sustained, and contested through the actions of people. The ‘motivation–incentive–opportunity’ triangle offers a valuable lens for understanding why actors behave differently across contexts, and how these behaviours influence the outcomes of policy formulation and implementation (Harris 2014).

In the context of health, the PE of health investigates how economic and political structures constrain or enable health behaviours and outcomes, interacting with personal, environmental, and institutional factors (Minkler et al. 1994, Harvey 2021) This approach critically addresses the social determinants of health—including socioeconomic status, race, class, and gender disparities—highlighting how these intersect with power relations to affect health inequalities and policy impacts (Minkler et al. 1994). It embraces an interdisciplinary, historical, and critical lens, examining health governance, policy framing, and the ethical dimensions of health systems (Minkler et al. 1994). As Lynch (2023) emphasizes, to understand the complex impacts of capitalism, neoliberalism, racism, and other systemic factors on health, PE must specify and contextualize these forces rather than treating them as vague or assumed determinants. This requires breaking down large institutions into their constituent mechanisms and tracing their influence on specific health outcomes. Achieving this level of analysis demands collaboration across political science, economics, and public health disciplines (Lynch 2023).

While a growing body of literature has examined the Political Economy of Health, the field remains theoretically rich but methodologically fragmented. Foundational contributions by scholars such as Navarro, Raphael, Schrecker, and Bryant (Navarro 2009, Schrecker and Bambra 2015, Bryant 2016, Raphael 2016) have provided robust critiques of capitalism, neoliberalism, and social inequities, illuminating the macro structural determinants of health. These works have shaped a critical tradition that interrogates how power relations, economic structures, and political ideologies condition population health and institutional responses. However, most of these contributions are located within high-level theoretical or empirical critiques of specific contexts and often do not provide clear methodological pathways for conducting PEA in health research.

Previous attempts to review the application of PEA in health have been limited. One umbrella review conducted in 2017 by McCartney et al. examined the impact of PE on population health (McCartney et al. 2019). The review findings underscored the favourable conditions of PE associated with improved health outcomes. However, the review exclusively concentrated on previously published systematic reviews, thus limiting the evidence regarding PE's application to health and its conceptualization, definition, and frameworks for analysing health outcomes (McCartney et al. 2019).

In the process of developing a political economy study on oral health care delivery in India, the present authors encountered significant challenges in identifying methodological frameworks and conceptual guidance for beginners. The existing literature was either too abstract, overly focused on macro-political critiques, or tailored to specific regional contexts, without offering transferable tools for practical analysis. This experience revealed a broader gap in the literature: although PEA is increasingly advocated for by global health bodies such as the WHO (Reich 2019), there is a notable absence of synthesized knowledge on how PEH is defined, what frameworks guide its application, which health issues are studied using this lens, and what outcomes or metrics are reported. Addressing this gap is critical not only for advancing scholarship but also for strengthening the capacity of health policy and planning to engage with the political and economic realities that shape reform.

## Methods

A scoping review was chosen as the most appropriate approach for this study as it allows us to synthesize and summarize evidence from various sources and gain a broad understanding of PE's role in health research. A protocol outlining the methodology for this scoping review was prepared and published in a peer-reviewed journal (Karuveettil et al. 2024). The review followed the JBI methodology for scoping reviews and reported according to the Preferred Reporting Items for Systematic Reviews and Meta-Analyses extension for Scoping Reviews (PRISMA-ScR) checklist (Supplementary Table S1) (Tricco et al. 2018, Peters et al. 2020).

### Deviations from scoping review protocol

Initially, the review aimed to search databases such as MEDLINE, Scopus, Web of Science, Cochrane Central, CINAHL, Embase, ProQuest, DynaMed, and grey literature sources, including Google Scholar and OAIster. However, due to limited access, Web of Science and DynaMed were excluded from the final search. To enhance the comprehensiveness of the review, JBI Evidence Synthesis and Epistemonikos were added as additional databases.

### Research questions

In this scoping review, we aim to address the following research questions:

1. How is the concept of the ‘political economy of health’ defined and applied within the context of health-related PEA?
2. What are the reported concepts and frameworks available to study political economy analysis of health?
3. What health problems are addressed, and how is PEA applied in understanding and addressing them?
4. What are the reported outcomes (and measures) used in the political analysis of health?

### Eligibility criteria

For this scoping review, there were no restrictions based on socio-demographic attributes or population size; studies involving any population group, including diverse age, gender, ethnicity, and socioeconomic status, were considered. The core concept of interest was the application of PEA to health, including but not limited to political positioning, stakeholder mapping, power dynamics, political feasibility, and the joint analysis of political and economic determinants. Studies were included if they explicitly applied a conceptual or analytical approach consistent with structured PEA. While we did not limit inclusion to any specific framework, those lacking a clear conceptual foundation or identifiable analytic structure were excluded. For articles on the PE of health systems, WHO building blocks were used for reporting outcomes, which include: (i) service delivery, (ii) health workforce, (iii) health information systems, (iv) access to essential medicines, (v) financing, and (vi) leadership/governance (Mounier-Jack et al. 2014).

The context was broad, covering various geographical areas and health concerns without any restrictions. PEA at both sector and country levels was examined for inclusion, along with studies involving multiple settings or cross-country comparisons. Although we intended to cover both health systems and population health, the final sample focused more heavily on health systems studies, reflecting how PEA is often labelled and structured in the health literature.

### Types of studies

This scoping review considered a wide range of studies without imposing restrictions on any specific study design and included an analysis of health intervention programs alongside other relevant investigations. Studies with statistical modelling analysis, not limited to regression model analysis, were also included. Opinion papers, narrative and systematic reviews were excluded. Conference abstracts and editorials without structured PEA were also excluded.

### Search strategy

A three-step search strategy was employed to locate both published and unpublished studies. Initially, a limited search of MEDLINE (Ovid) was conducted to have an overview of the literature, followed by an examination of the text words and index terms related to the political economy of health. The initial keywords identified are ‘political economy’ ‘political systems’ ‘health politics’ ‘power relationships’ ‘public finance’ ‘transnational corporations’ ‘welfare states’ ‘health financing’ ‘health systems’ ‘health reform’ ‘health governance’ ‘health policy’ ‘public policy’ and ‘social determinants of health’. Using this information, a customized search strategy was devised and adjusted for each database.

Databases searched included MEDLINE (Ovid), Scopus, Cochrane CENTRAL, CINAHL (EBSCO), JBI Evidence Synthesis (Ovid) and Epistemonikos as well as grey literature sources such as Google Scholar, ProQuest Dissertations and Theses (ProQuest), OAIster, and government reports or documents from civil society or international health organisations related to health policy analysis. The full search strategy was initially conducted on 20 February 2023, and subsequently updated on 31 October 2024, and 5 May 2025. A reference list of all included studies (following full-text screening) was reviewed for additional studies, but no further studies were identified. The review considered all relevant studies, with no restrictions on language or publication dates. Supplementary Table S2 details the search strategy across all published and un-published literature.

### Study/source of evidence selection

Following the search process, all identified records were gathered and organized using Mendeley v.2.64 (Mendeley Ltd., Elsevier, Netherlands), and duplicate entries were removed. Two reviewers independently conducted the screening of titles and abstracts, evaluating records against the predefined inclusion criteria for the review. Potentially relevant studies underwent complete retrieval, and their citation details were imported into the JBI System for the Unified Management, Assessment, and Review of Information (JBI SUMARI; JBI, Adelaide, Australia) (Munn et al. 2019). The full texts of selected studies were retrieved and thoroughly assessed against the inclusion criteria by two reviewers. In case of any discrepancies between the reviewers, a third reviewer was consulted for resolution. Following the screening and identification of studies, the reference lists of included studies were manually searched for relevant citations.

### Data extraction

Two independent reviewers performed data extraction using a customized data extraction tool (Table 2). Data extraction was performed in JBI SUMARI with any discrepancies between the two reviewers resolved by a third reviewer. The data extraction form gathered details including the study title, the health problem or issue in question, the adopted definition of political economy, the theoretical or conceptual background utilized for PEA, the adopted political-economic framework, significant findings or factors identified for the health issue using PEA, and any additional information pertinent to PEA.

### Data analysis and presentation

The study selection and inclusion process is displayed following the PRISMA 2020 flow diagram (Tricco et al. 2018). This review used various methods to synthesise and summarize the results, which are illustrated through tables and figures. Review findings are presented as answers to each research question. A tabular format was used to organize the review findings, accompanied by a narrative description. A word cloud was created using the keywords from the adopted definitions. At all stages of the study, stakeholders in health policymaking (a minimum of five experts) were invited to participate in discussions. The initial results were discussed among the authors of the review and relevant stakeholders (including two political economy experts, two health economics experts, and one health policymaker). Any relevant inputs provided were incorporated in the final presentation of results below.

## Results

### Study inclusion

**Figure 1.**
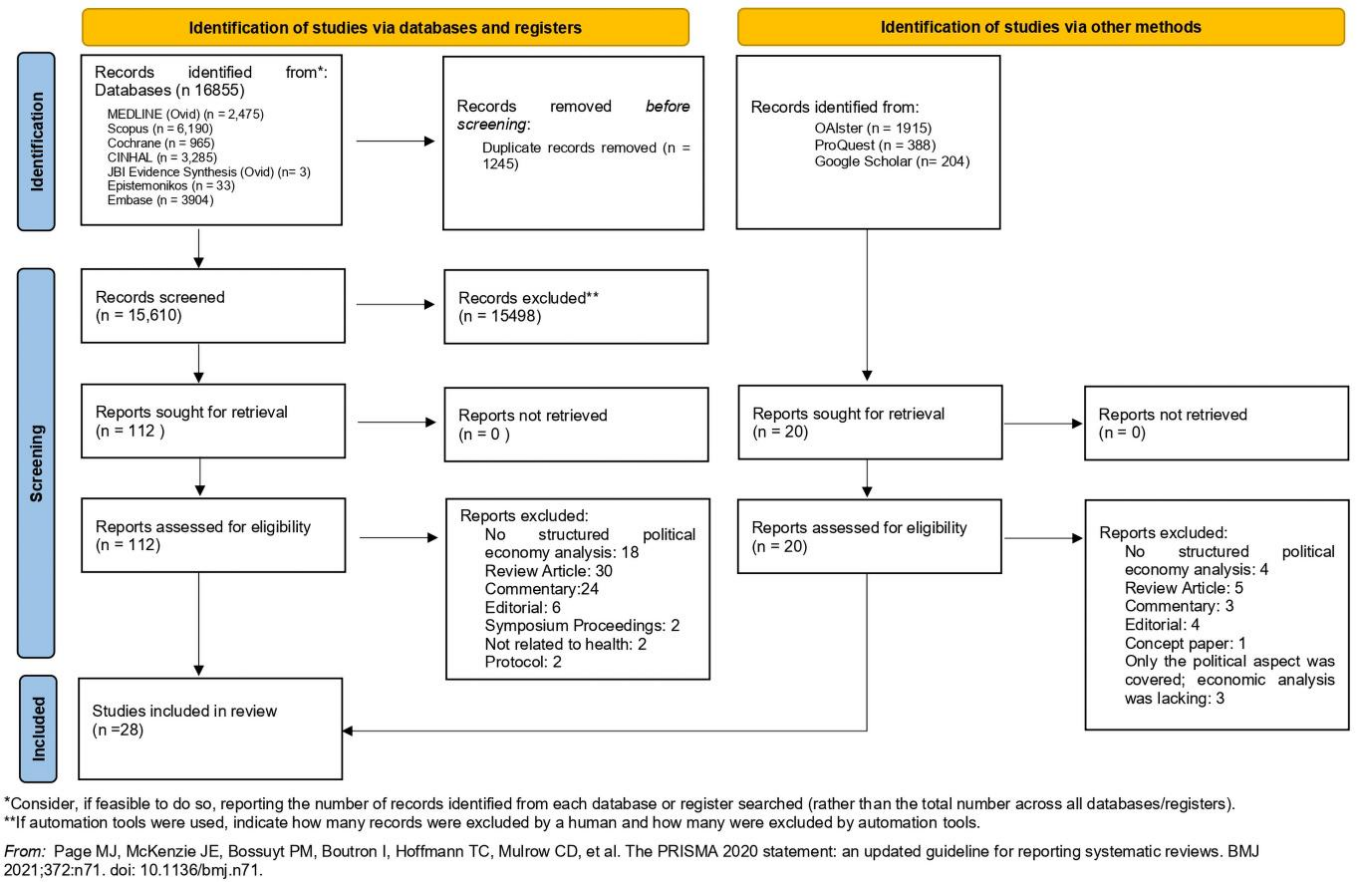
Flow diagram of the study selection process according to PRISMA guidelines.

The search strategy across databases yielded 19 362 records, and after removing 1245 duplicates, 18 117 records were screened by two independent reviewers based on titles and abstracts. Of these, 16 985 records were excluded based on the inclusion and exclusion criteria. The same reviewers performed full-text screening on 122 articles, and 28 articles were selected for inclusion for review and analysis. Figure 1 illustrates the search and selection process. The list of excluded studies after full-text screening is reported in Supplementary Table S3.

### Characteristics of included studies

Of the 28 studies included in the review, the year of publication ranged from 2015 to 2025 (Supplementary Figure S1). The review covered a wide range of geographical regions, spanning 30 countries (Supplementary Figure S2), including conflict-affected areas such as South Sudan (Widdig et al. 2022), Sierra Leone (Bertone and Witter 2015), and the Democratic Republic of the Congo (Bigirinama et al. 2024). Other African nations represented included Madagascar (Nonvignon et al. 2023), Malawi (Nonvignon et al. 2023, Rodríguez et al. 2023), Uganda (Bukenya and Golooba-Mutebi 2020, Nannini et al. 2021), Kenya (Rodríguez et al. 2023, Tsofa et al. 2023), Comoros (Nonvignon et al. 2023), Zimbabwe (Witter et al. 2019, Nonvignon et al. 2023), Ethiopia (Fieno et al. 2016, Nonvignon et al. 2023), Tanzania (Kuwawenaruwa et al. 2023), Nigeria (Croke and Ogbuoji 2024, Chukwuma and Obi 2025) and Burundi (Nonvignon et al. 2023). From the Americas, studies were conducted in Mexico (Gómez-Dantés et al. 2015, Sparkes et al. 2019), Chile, Canada (Sing et al. 2025) and the United States (Fox and Choi 2019, Fujishiro et al. 2021). Studies from Asian countries included Bangladesh (Hipgrave et al. 2019), Indonesia (Ahsan et al. 2024, Hipgrave et al. 2019), Nepal (Hipgrave et al. 2019), Turkey (Sparkes et al. 2019), Afghanistan (Salehi et al. 2021), Thailand (Tangcharoensathien et al. 2019), Malaysia (Barraclough and Morrow 2017, Croke et al. 2019), Timor-Leste (Bertone et al. 2018), and the Philippines (Hipgrave et al. 2019). Oceania countries included were Fiji (Thow et al. 2021, Mounsey et al. 2022) and Tonga (Mounsey et al. 2022). One study was included from the European Union (Fujishiro et al. 2021) and from United Kingdom (Sing et al. 2025).

The studies included in this review employed a variety of study designs, which in many cases were combined to perform political economy analysis, as shown in Table 1. The majority of the studies, twenty in number, used qualitative approaches (Fieno et al. 2016, Bertone et al. 2018, Croke et al. 2019, Fox and Choi 2019, Hipgrave et al. 2019, Sparkes et al. 2019, Witter et al. 2019, Bukenya and Golooba-Mutebi 2020, Nannini et al. 2021, Salehi et al. 2021, Thow et al. 2021, Mounsey et al. 2022, Widdig et al. 2022, Kuwawenaruwa et al. 2023, Nonvignon et al. 2023, Tsofa et al. 2023, Ahsian et al. 2024, Bigirinama et al. 2024), including descriptive qualitative methods (Witter et al. 2019, Nannini et al. 2021, Salehi et al. 2021, Kuwawenaruwa et al. 2023, Nonvignon et al. 2023, Ahsan et al. 2024) and case studies (Fieno et al. 2016, Sparkes et al. 2019, Witter et al. 2019, Bukenya and Golooba-Mutebi 2020, Nannini et al. 2021, Thow et al. 2021, Mounsey et al. 2022, Widdig et al. 2022, Tsofa et al. 2023, Bigirinama et al. 2024). Secondary data analysis in the form of eight literature reviews (Gómez-Dantés et al. 2015, Fieno et al. 2016, Croke et al. 2019, Hipgrave et al. 2019, Tangcharoensathien et al. 2019, Nannini et al. 2021, Salehi et al. 2021) and eleven document analyses (Barraclough and Morrow 2017, Bertone et al. 2018, Fox and Choi 2019, Tangcharoensathien et al. 2019, Witter et al. 2019, Nannini et al. 2021, Salehi et al. 2021, Mounsey et al. 2022, Widdig et al. 2022, Kuwawenaruwa et al. 2023, Bigirinama et al. 2024), Norris (2025) provided insights into historical trajectories and policy influences. Five studies employed mixed methods (Bertone and Witter 2015, Gómez-Dantés et al. 2015, Bertone et al. 2018, Hipgrave et al. 2019), combining qualitative and quantitative elements, while one utilized a longitudinal survey (Bertone and Witter 2015). In-depth stakeholder analyses, involving process tracing and policy analysis, were reported in three studies (Croke et al. 2019, Fox and Choi 2019, Mounsey et al. 2022). The majority of the data collection was performed using key informant interviews (Bertone et al. 2018, Witter et al. 2019, Bukenya and Golooba-Mutebi 2020, Nannini et al. 2021, Thow et al. 2021, Widdig et al. 2022, Kuwawenaruwa et al. 2023, Nonvignon et al. 2023, Rodríguez et al. 2023, Tsofa et al. 2023, Bigirinama et al. 2024). The studies often utilized a combination of these methods, such as mixed-method research incorporating literature review, field interviews, and policy analysis (Fox and Choi 2019, Hipgrave et al. 2019, Mounsey et al. 2022).

**Table 1.** Characteristics of included studies

Study ID (Author, year)	Country	Health problem/Issue	Study design and data collection	Data analysis	Outcomes identified	Outcomes classified according to WHO building blocks
**Salehi et al. (2021)**	Afghanistan	Performance-Based Financing in Afghanistan	Retrospective qualitative research and desk review of Government reports/Document analysis	Content Analysis	Influence of global policy context, implementation experience in other LMICs, power dynamics and interaction between actors	Financing
**Bertone and Witter (2015)**	Sierra Leone	Human Resource for Health (HRH) Incentive implementation and informal practice in West Africa	Mixed Method Research Key informant interview Longitudinal survey	Thematic analysis	Influence of structural features, context, institutions, main actors with their activities and agendas, actor relationships and their balance of power	Health Workforce, Financing, Governance
**Sparkes et al. (2019)**	Turkey and Mexico	Health Financing reform processes of two countries. Turkey’s Transformation in Health Program (2002–2012) and Mexico’s Seguro Popular (2000–2006).	Case study. Political economy framework application	Retrospective analysis of health financing reform experiences	Health financing reform is an inherently political process. Political and technical actors are closely intertwined.	Governance, Financing
**Fieno et al. (2016)**	Ethiopia	Human resource for health crisis in Ethiopia	Literature Review and Case study	Political economy analysis at multiple levels	Political will coupled with strong state capacity and adequate resource mobilization can overcome the institutional hurdles	Health Workforce
**Bertone et al. (2018)**	Timor-Leste	Human resources for health (HRH) recruitment in post conflict setting (Timor-Leste)	Mixed Method Literature/Document search along with key informant interviews.	Policy analysis and thematic analysis	Influence of national and international actors, lack of intersectoral co-ordination. Difficulty of reconciling the technocratic with the social, cultural and political concerns.	Health Workforce
**Tsofa et al. (2023)**	Kenya	Sub-national level health sector governance following public sector decentralization (Devolution) in Kenya	Case study, Key informant interview	Framework analysis approach, Thematic analysis	National health sector priority agenda issues, local political interests, equity considerations, and the interactions among various actors involved in the priority-setting process are identified as key drivers of county-level processes.	Financing, Governance
**Hipgrave et al. (2019)**	Bangladesh, Indonesia, Nepal and the Philippines	District-level health and MNCH (Maternal, New-born and Child health) financing and performance in LMIC	Mixed method research. Literature review, field interviews, policy, process and budget analysis.	Thematic analysis	Apparent political commitment to MNCH manifests in a variety of ways, ranging from local or national health authorities influenced by larger national political and economic issues	Finance, Health Workforce, Governance
Gomez-Dantes (2015)	Mexico	Social Protection Reform Adoption Process in Health (Mexico)	Mixed Method research. Academic literature review and direct interviews.	Context and stakeholder analysis	Powerful actors can exploit favourable circumstances to shape and implement SPH policies without needing citizen mobilization.	Governance
**Bukenya and Golooba-Mutebi (2020)**	Uganda	Sub-National Variation in Maternal Mortality Rates in Uganda	Comparative case study design. Key informant interviews, focus group discussions (FGD) and observation.	Thematic analysis	Informal institutional context in which public service delivery operates largely explains sub-national differences in maternal mortality	Governance
**Witter et al. (2019)**	Zimbabwe	Results-based financing (RBS) in the health sector in Zimbabwe	Retrospective qualitative case study. Key informant interviews and analysis of policy documents	Thematic analysis	Local health system actors can shape and adapt RBF to suit their needs in the light of limited resources. RBS is functioning as the main provider payment mechanism for under-funded primary care.	Finance
**Mounsey et al. (2022)**	Fiji and Tonga	Sugar Sweetened Beverage (SSB) taxation for Non Communicable Disease Prevention in Fiji and Tonga	Comparative case study. Documentary policy and stakeholder analysis with semi-structured stakeholder interview.	Policy documentary analysis, comparative analysis and thematic analysis	Multisectoral engagement, a whole-of-society approach, strengthened institutions, and leveraging competing priorities could increase SSB tax impact towards common goals.	Governance
**Widdig et al. (2022)**	South Sudan	Priority-setting practices in the Health Pooled Fund (HPF) for health service delivery in South Sudan	Retrospective case study approach and critical realist approach. Key informant interviews, document review and quantitative examination of service delivery	Thematic analysis	Donor dominance in the priority-setting process, fuelled by power asymmetries with national health authorities, restricts government ownership, leaving little room for their influence.	Service Delivery, Finance
**Thow et al. (2021)**	Fiji	Restricted marketing of unhealthy food and breast milk substitutes for children in Fiji	Case Study approach. Policy analysis and Key informant interviews	Thematic analysis	Policy landscape is shaped by a new paradigm, limited awareness among key actors on nutrition-marketing links, and a power imbalance between industry and health stakeholders.	Governance
**Fox and Choi (2019)**	USA	Politics of health reform (New York health act: single payer plan) in New York state to create universal health financing plan	Process tracing approach. Evidence from policy documents, transcripts of public hearings, media reporting and interviews with stakeholders involved in the policy process.	Framework analysis	A clear political opportunity is in place for single-payer plan, but the prospects for adoption remain low given the power of symbolic politics and institutional inertia on the reform process.	Finance
**Croke et al. (2019)**	Malaysia	Failure of adoption of health financing reform in Malaysia	In-depth stakeholder analysis using semi-structured interviews and literature review.	Stakeholder analysis, Veto gates analysis, and Historical institutionalist analysis	Historical institutionalist approach best explains Malaysia’s persistently blocked reforms, while interest groups mobilization played an important secondary role.	Finance
**Nannini et al. (2021)**	Uganda	Ugandan experience of the abolition of user fees in public health facilities and the debate on National Health Insurance	Qualitative research involving, desk review and Key informant interview (semi-structured interview guide).	Constant comparison analysis.	Political economy conditions are not yet favourable for universal health coverage; in particular, interests and ideas are not conducive to expanding financial protection.	Finance
**Fujishiro et al. (2021)**	USA, European Union	Framework development for research on work, health, and health equity in institutional contexts. (United States and European Union)	Framework development and application	Framework analysis	Understanding working people’s health with a clear awareness of its institutional contexts is imperative for developing policy interventions.	Workforce
**Barraclough and Morrow (2017)**	Malaysia	The political economy of tobacco within Malaysia's politically dominant ethnic group.	Primary and secondary data analysis of relevant documents	Secondary data analysis (Descriptive)	Ethnicity, a pivotal factor in Malaysian political economy, has been overlooked in tobacco control research and policy discussions	Governance
**Tangcharoensathien et al. (2019)**	Thailand	Universal Health Coverage Reform in Thailand	Desk review	Path dependency and Framework analysis	Understanding the political economy dimensions of reform, the power and position of different actors, the culture of using evidence for decisions and leadership are enabling factors for successful reforms.	Finance
**Bigirinama et al. (2024)**	Congo	Maternal and new-born health (MNH) prioritization in the Congo	Descriptive case study using in-depth interviews with key actors at various health system levels and desk review.	Thematic analysis	MNH policy implementation is challenged by health and security crises, limited resources, governance issues, and gender disparities.	Governance
**Nonvignon et al. (2023)**	Burundi, Comoros, Ethiopia, Madagascar, Malawi and Zimbabwe.	Reliance on donor funding for traditional vaccines and vitamin A supplementation	Qualitative study with semi-structured interviews with key informants.	Thematic analysis	Enablers for financing: emerging reforms, citizen voice, improved domestic economy. Barriers: political instability, health sector inefficiencies, complex bureaucracy, leadership turnover, competing priorities.	Finance, Service Delivery
**Rodríguez et al. (2023)**	Kenya, Malawi and Uganda	Subnational health management	Embedded multiple case study design with data collected through document review and key informant interviews.	Framework Method thematic analysis and, where available, quantitative analysis of financial and spending trends.	Key challenges included persistent bureaucratic barriers, budget constraints, weak accountability with development partners, inconsistent community engagement, limited local administrative capacity, and the need for better communication and flexible funding from the central government.	Governance
**Ahsan et al. (2024)**	Indonesia	Health tax policy implementation for tobacco, alcohol, and sugar sweetened beverage	Qualitative study with semi structured focus group discussions.	Framework analysis using thematic coding	Cultural factors drive tobacco and alcohol use; low awareness of SSB health risks; bureaucratic complexity and decentralization hinder implementation of tax; need for clear policy communication and earmarked tax revenue for public health.	Governance
**Kuwawenaruwa et al. (2023)**	Tanzania	Health Management Information System (HMIS) strengthening	A qualitative study incorporating data collection through document review and key informant interviews.	Thematic analysis	Key drivers included data needs, strong government-stakeholder relationships, and long-term partnerships influencing investment decisions; barriers included lack of technical staff, financial constraints, and fragmented systems.	Governance, health information system
**Chukwuma and Obi (2025)**	Nigeria	Health system reform and universal health coverage (UHC); role of private sector in healthcare delivery	Political economy analysis using interviews (*n* = 52) and FGDs (*n* = 12); historical and document analysis (1950s–2014)	Qualitative thematic analysis	Path-dependent reliance on private healthcare Limited earmarking for UHC in NHAct Institutional distrust in public system	Governance, Finance, Service Delivery, Workforce
**Croke and Ogbuoji (2024)**	Nigeria	Implementation of two key reforms: Saving One Million Lives (SOML) & National Health Act (NHA)	Within-case comparative design; 23 stakeholder interviews; policy and literature review	Thematic analysis; informed by political settlements theory and policy cycle model	Horizontal and vertical governance fragmentation Weak institutional accountability Limited reform sustainability post-election turnover	Governance, Finance, Health Information Systems, Service Delivery
**Norris (2025)**	USA (Washington State)	Barriers to dental care access; Initiative 678 to allow independent practice by dental hygienists	Quantitative analysis of 1997 voting data across 39 counties; demographic, health, and professional density data	Median voter model incorporating special interest variables	Voters in underserved areas more likely to support reform Initiative failed due to lobbying by dentists	Service Delivery, Health Workforce, Governance
**Sing et al. (2025)**	Chile, Canada, United Kingdom	Unhealthy food and beverage marketing to children	Multiple case study; documentary evidence and 21 semi-structured key informant interviews	Political economy analysis using the 3Is framework (institutions, interests, ideas) and associated power dynamics	Neoliberal institutional norms constrained government action Strong mandates and policy entrepreneurs crucial for legislative success Role of discursive framing (e.g. child protection, public health necessity) Industry resistance through lobbying and discursive strategies	Governance

### Review findings

This scoping review aimed to identify the concepts, definitions, frameworks, outcomes, and applications of political economic analysis of health.

### Review question 1: How is the concept of the ‘political economy of health’ defined and applied within the context of health-related PEA?

#### How was the political economy defined?

The studies included in this review reported a range of definitions for political economy. Among the twenty-four studies examined, only eleven (Bertone and Witter 2015, Gómez-Dantés et al. 2015, Fieno et al. 2016, Bertone et al. 2018, Fox and Choi 2019, Hipgrave et al. 2019, Sparkes et al. 2019, Witter et al. 2019, Fujishiro et al. 2021, Salehi et al. 2021, Mounsey et al. 2022, Nonvignon et al. 2023) offered a clear and specific definition of PE (Table 2). Some studies provided definitions sourced from existing literature (Bertone and Witter 2015, Gómez-Dantés et al. 2015, Fieno et al. 2016, Bertone et al. 2018, Hipgrave et al. 2019, Witter et al. 2019, Fujishiro et al. 2021, Mounsey et al. 2022, Nonvignon et al. 2023), while others amalgamated multiple definitions to outline the essence of PE (Fox and Choi 2019, Sparkes et al. 2019, Salehi et al. 2021). However, a few studies did not distinctly provide a clear definition (Barraclough and Morrow 2017, Croke et al. 2019, Tangcharoensathien et al. 2019, Bukenya and Golooba-Mutebi 2020, Nannini et al. 2021, Thow et al. 2021, Widdig et al. 2022, Kuwawenaruwa et al. 2023, Rodríguez et al. 2023, Tsofa et al. 2023, Ahsan et al. 2024, Bigirinama et al. 2024). Political economy encompasses the study of power dynamics, resource distribution, and interactions among various actors shaping policies and programs (Bertone and Witter 2015, Gómez-Dantés et al. 2015, Fieno et al. 2016, Witter et al. 2019, Salehi et al. 2021, Mounsey et al. 2022). It examines the structural context (Bertone et al. 2018), political feasibility of reforms (Sparkes et al. 2019), and the roles of institutions at both domestic and international levels (Fieno et al. 2016, Witter et al. 2019). PE also considers the influence of political actors, stakeholders, and donors on decision-making processes, resource allocation, and policy trajectories (Bertone et al. 2018, Hipgrave et al. 2019, Sparkes et al. 2019, Mounsey et al. 2022). It explores the interrelations between political and economic environments, state capability, accountability, and responsiveness within policy cycles (Mounsey et al. 2022). In health contexts, PE analyses political factors affecting health financing reforms towards universal coverage (Fox and Choi 2019), and examines how macro-level structural determinants shape behavioural and social determinants of health (Fujishiro et al. 2021). A summary of these definitions highlights the diversity in PE conceptualizations across the studies (Table 2). Although the definitions varied across studies, a common thread that emerges is the conceptualization of political economy as a dynamic framework that analyses how power, institutions, and interests shape policy processes and health outcomes. A word cloud was created to visualise prominent keywords used in defining PE (Supplementary Figure S3).

**Table 2.** Details of political economy applications in health

Study ID (Author, year)	Political economy definition	Rationale for using PEA	Theoretical/conceptual backing	Framework used
**Salehi et al. (2021)**	Political economy analysis (PEA), studies power and resource distribution and contestation, the roles played by different actors and their interactions, and how this shapes programmes and policies (Poole 2011, Reich 2019).	For understanding factors (context, actors, processes) influencing the PBF adoption, design and implementation.	Contextual factors, power dynamics and path dependency	Policy Engagement Framework by Buse et al. (2008)
**Bertone and Witter (2015)**	Dynamics between the layers of the structural context and the multiple actors and organisations that shape practices (Harris 2014).	To analyse how context, actors and organizations influence HRH policy implementation.	Agency Theory Stewardship theory (Perrow 1986) (Economic theory)	Applied political economy framework b Harris (2014)
**Sparkes et al. (2019)**	Political economy analysis is used to assess the power and position of key political actors, as a way to develop strategies to change the political feasibility of desired reforms (Whaites 2017; Menocal et al. 2018).	To understand reform experiences and its potential for helping decision-makers manage reform processes prospectively. To report the importance of political economy factors in determining policy trajectories.	Interest group politics, bureaucratic politics, budget politics, leadership politics, beneficiary politics, and external actor politics.	The Political Economy of Health Financing Reform Framework by Campos and Reich (2019)
**Fieno et al. (2016)**	An analysis of institutions or structures that shape HRH policy, namely, the political environment, domestic institutions, and international structures Hudson 2014).	To understand how policy is made, how leaders are accountable, how international structures encourage (or distort) health policy, and how development objectives are prioritized in these countries.	Application of economic thinking to politics.	Institutional political economy approach (Hudson 2014).
**Bertone et al. (2018)**	Elements of the agency and structure contributed to drive the policy trajectory (Harris 2014).	To explore how and why both official and informal practices developed, as well as the drivers, challenges and blockages at different stages of health workforce recruitment.	Agency (actors, agendas, power relations) and structure (socio economic conditions, historical legacies, formal and informal institutions, cultural norms)	Applied political economy framework b Harris (2014)
**Tsofa et al. (2023)**	Authors state there is no universal definition or framework for conducting PEA	To understand how planning and budgeting processes are structured, enacted, and subject to change for sub-national health management.	Structural Diagnosis (Context and Institutions) and Agency Diagnosis (Power, Incentives and Behaviour)	Adapted framework of Problem driven Political Economy Analysis by Siddiqi et al. (2009)
**Hipgrave et al. (2019)**	Stakeholders can be explicitly or implicitly involved in economic processes that influence resource allocation (DFID 2009).	To investigate how four Asian LMICs (Bangladesh, Indonesia, Nepal and the Philippines) allocate and utilize resources for maternal, newborn and child health (MNCH).	‘Theory of change’, ‘drivers of change’, ‘most significant change	Multiple frameworks including PEA prepared by the UK DFID (DFID 2009) and the Poole (2011), an approach developed by the Overseas Development Institute) (Harris et al. 2014) and the World Bank’s ‘problem-driven governance’ framework (Fritz et al. 2009).
Gomez Dantes (2015)	‘A variety of approaches for studying economic and political behaviour, and the understanding of how political institutions, social and political power relations, and the political and economic environment influence each other’.	To identify the factors that contributed to the adoption of policies to expand social protection in health (SPH).	The Policy Circle framework by Hardee et al. Enabling and driving factors Andersen’s behavioural mode of families (Andersen 1968).	Multiple models of The Policy Circle framework by Hardee et al. (2004) Andersen’s behavioural model (Andersen 1968) and Stakeholder analysis by Varvasovszky and Brugha (2000)
**Bukenya and Golooba-Mutebi (2020)**	No clear definition is provided	To explain sub-national differences in Maternal Mortality Rates.	Political settlement theory by Khan (Mushtaq 2009)	Accountability framework by World Bank (Agnès 2004) and Political settlement theory by Khan (Mushtaq 2009)
**Witter et al. (2019)**	How formal and informal institutions influence and are influenced by decision-making, power and resources (Harris 2014).	To understand the influence of the starting context, institutions and actors on the Results-based financing development and adoption, and how the programme shaped these factors.	Interaction of actors, context and resources (power and financing).	PEA prepared by the UK Department for International Development (DFID) (DFID 2009)
**Mounsey et al. (2022)**	Understanding of the power, state capability, accountability and responsiveness of the stakeholders and donors involved in a policy cycle (DFID 2009).	To identify the relationships and interactions, interests, incentives, cultural and political constraints to, or opportunities for, shaping and strengthening policy implementation and accelerating progress for improved nutrition	Shiffman’s framework for global health policy priority setting (Shiffman and Smith 2007), Kingdon’s ‘multiple streams’ theory (Kingdon 1995)	Shiffman’s framework for global health policy priority setting (Shiffman and Smith 2007), Kingdon’s ‘multiple streams’ theory (Kingdon 1995)
**Widdig et al. (2022)**	No clear definition is provided	To capture and describe the practices and experiences that define the priority-setting landscape through the examination of the Health Pooled Fund (HPF).	Context, content, process and actors. Concepts of institutions, interests, and Ideas.	Adapted version of the Walt and Gilson (1994) policy analysis triangle (Walt and Gilson 1994).
**Thow et al. (2021)**	No clear definition is provided	What influences decisions on restricting the marketing of foods to (for) children?	Policy theory, Priority Setting	Political Economy Framework by Campos and Reich (2019)
**Fox and Choi (2019)**	The Political Economy of Health Financing Reform Framework draws on observations regarding the politics of health financing reform. The framework takes the vantage point of the health reform change team and examines common political factors faced by reform teams whose goal is to reform health financing towards universal coverage (Campos and Reich 2019; Harvey 2021).	Why has the United States continued to rely on private, employer-sponsored coverage and failed at attempts to unify its health coverage system into a universal financial protection for all? If this cannot be achieved at a federal level, can individual states do better?	Interest group politics; leadership politics; budget politics; bureaucratic politics; beneficiary politics; and external politics.	Political Economy Framework by Campos and Reich (2019)
**Croke et al. (2019)**	No clear definition is provided	To examine why health financing reforms were continuously proposed by the government but consistently blocked by opponents.	Interest group-centred theory (Rajan and Zingales 2003), Veto gates theory (Immergut 1992), historical institutionalist theory (Pierson and Skocpol 2002) (Path dependency and Policy feedback)	Interest group-centred theory (Rajan and Zingales 2003), Veto gates theory (Immergut 1992), historical institutionalist theory (Pierson and Skocpol 2002), stakeholder analysis by Reich (1996) and Stakeholder analysis by Varvasovszky and Brugha (2000).
**Nannini et al. (2021)**	No clear definition is provided	To examine the impact of health financing reforms, specifically the abolition of user-fees in public health facilities and the debate on National Health Insurance, on financial protection in the healthcare system in the last two decades in Uganda.	Stakeholder and Institutions, Politics (Negotiation on reforms), Policy (Implementation of reforms), coverage outcome and health outcomes	The Political Economy of Health Financing Reform Framework by Campos and Reich (2019), Shiffmans framework (Shiffman and Smith 2007)
**Fujishiro et al. (2021)**	‘The behavioral and social determinants of health are themselves shaped by macro-level structural determinants […] the structures, values, and priorities of political and economic systems’ (Bambra 2019)	To examine work as a social determinant of health and health inequity with an explicit focus on how it is embedded in social institutions.	Social institutions, social values, theory of discursive institutionalism and sorting systems	Macro-level Model of Employment Relations (Muntaner et al. 2010), Social basis of disparities in health (Diderichsen et al. 2001)
**Barraclough and Morrow (2017)**	No clear definition is provided	To identify the historical nexus between Malaysia’s largest and politically dominant ethnic group and the political economy of tobacco, and to consider the implications of this connection for tobacco control.	No clear theories or concepts provided	No clear framework mentioned
**Tangcharoensathien et al. (2019)**	No clear definition is provided	How political economic factors influenced the evolution of the UHC reform in Thailand	Path Dependency (Pierson 1993; Haeder 2012)	Path Dependency (Pierson 1993; Haeder 2012)
**Bigirinama et al. (2024)**	No clear definition provided	To understand the complex factors that facilitate or hinder the MNH policy prioritization, development, and implementation in 2 provinces in Congo.	Context, content, process and actors. Concepts of institutions, interests, and ideas.	Adapted version of the Walt and Gilson (1994) policy analysis triangle.
**Nonvignon et al. (2023)**	No clear definition provided	To understand the reasons donors and governments continue to depend on external funding for traditional vaccines and Vitamin A supplementation.	Leadership politics; budget politics; bureaucratic politics; external actor politics; External environment.	The Political Economy of Health Financing Reform Framework by Campos and Reich (2019) and Sparkes et al. (2019)
**Rodríguez et al. (2023)**	No clear definition provided.	To understand how local governance, power dynamics, and bureaucratic barriers impact health managers’ ability to improve PHC access and quality.	Structural Diagnosis (Context and Institutions), Agency Diagnosis (Power, Incentives and Behaviour) and pathways of change.	Adapted framework of Problem driven Political Economy Analysis by Siddiqi et al. (2009)
**Ahsan et al. (2024)**	No clear definition provided.	To identify challenges and opportunities in implementing health taxes at the sub-national level in Indonesia.	Structural and agency issues in policy execution.	Problem-driven political economic analysis adapted from the Overseas Development Institute (Agnès 2004)
**Kuwawenaruwa et al. (2023)**	No clear definition provided.	To analyse relationships, power dynamics, and drivers/barriers affecting HMIS investment and strengthening in Tanzania.	Structural Diagnosis (Context and Institutions), Agency Diagnosis (Power, Incentives and Behaviour) and pathways of change.	The adapted framework of Problem driven Political Economy Analysis by Siddiqi et al. (2009)
**Sing et al. (2025)**	Political economy is framed through institutions, interests, and ideas to explain how power operates across political systems when legislating for public health.	To understand barriers and enablers to laws restricting unhealthy food marketing to children in Chile, UK, and Canada.	Grounded in Pettit & Mejía Acosta’s power analysis (Pettit 2013) Lukes’ (Lorenzi 2006) Gaventa’s power theories (Gaventa 2006) and political science literature on institutions, actor power, and discursive framing.	Political Economy Analysis Framework (3Is: Institutions, Interests, Ideas) + Power Dimensions
**Chukwuma and Obi (2025)**	Political economy is used to understand how ideologies, interests, and institutions influence universal health coverage (UHC) reforms.	To investigate how these factors shaped the Nigerian health system reforms, particularly the 2014 NHAct and role of the private sector.	Based on pluralist, institutional, development, and class theories; draws on path dependency and historical institutionalism.	Rizvi et al. (2020) framework based on Fox and Reich (2015)’s ‘4 Is’ (Interests, Institutions, Ideas & Ideology) (Rizvi et al. 2020)
**Croke and Ogbuoji (2024)**	Political economy considers how policy elites’ ideas and training interact with political context and competing interest groups.	To explain adoption and implementation of UHC reforms in Nigeria, including SOML and the 2014 National Health Act.	Combines the politics of policy reform model (Grindle and Thomas 1991) and political settlements (Khan 1995; Kelsall et al. 2016)	Policy Cycle Model + Political Settlement Analysis (PSA) (Khan 1995)
**Norris (2025)**	Political economy analyses how rent-seeking and institutional structures shape direct democracy initiatives, particularly where scope-of-practice laws are contested.	To study how special interest groups and voter self-interest shaped support for Initiative 678 in Washington state.	Median voter model, public choice theory (Buchanan and Tullock 1965) and democratic responsiveness; compares legislative vs. direct democracy processes.	Median Voter Model with Special Interest Variables (Black 1948)

### Review question 2: What are the reported concepts and frameworks available to study political economy analysis of health?

#### Framework used

The review identified 31 unique frameworks or concepts for PEA from the twenty-four included studies (Table 3). The most frequently employed framework was the ‘The Political Economy of Health Financing Reform Framework’ developed by Campos and Reich (Sparkes et al. 2019) which was used in studies exploring health financing, regulatory policies, and health reform. ‘Applied political economy framework’ by Harris et al (Harris 2014) is another widely used approach to examine issues related to human resources for health and health financing. Studies utilized step by step frameworks such as ‘How to’ notes on Political Economy Analysis prepared by the UK’s Department for International Development’ (DFID 2009), ‘HOW-TO NOTES Political Economy Assessments at Sector and Project Levels’ (Poole 2011), ‘Policy Engagement Framework’ (Buse et al. 2008), ‘Problem-driven GPE analysis’ (Fritz et al. 2009 ), and ‘Applied political analysis for health policy reform’ (Reich 1996), focus on health financing. Theories like ‘Veto gates theory’ (Immergut 1992) ‘Historical institutionalist theory’ (Pierson and Skocpol 2002 ) and ‘Interest Group Theory’ (Rajan and Zingales 2003) are also used to understand how political and economic forces influence health financing. Studies focusing on governance predominantly employed frameworks such as the ‘Health system governance framework’ (Foley 1999 ), the ‘Accountability framework’ (Ahmad 2003) from the World Bank, and the ‘Political settlement theory’ (Khan 2010) by Mushtaq H. Khan. Walt and Gilson's policy triangle (Walt and Gilson 1994 ) The Policy Circle framework (Hardee et al. 2004 ) Stakeholder analysis (Varvasovszky and Brugha 2000). The Andersen Behaviour Model (Andersen 1968), and Path Dependence (Haeder 2012; Pierson 2004) are prominent frameworks used to analyse the various factors influencing health reform initiatives. The Political Settlement Analysis (PSA) (Khan 1995, Kelsall et al. 2016) has also been increasingly integrated into PEA. PSA focuses on how elite bargains and distributions of power influence institutional performance and policy implementation. Other frameworks include the ‘Institutional Political Economy Approach’ (Hudson 2014) for human resources for health, ‘Shiffman’s framework for global health policy’ (Shiffman and Smith 2007), ‘Kingdon’s ‘multiple streams’ theory’ (Kingdon 1995) on regulatory policies, and the ‘Macro-level Model of Employment Relations’ (Muntaner et al. 2010) and ‘Social Basis of Disparities in Health’ (Diderichsen et al. 2001) for health equity. Additional frameworks and theories found in the included studies reflect the evolving complexity of the PEA Integrated Political Economy Framework (2020) (Rizvi et al. 2020) emphasizes the dynamic interaction of ideas, interests, and institutions and is derived from the ‘Four Is’ model (Fox and Reich 2015). 3Is + Power Framework (Pettit and Mejia Acosta 2014) extends traditional PEA by incorporating actor, institutional, and discursive power.

**Table 3.** Frameworks used in political economy in health

S. no	Framework	Year	Author	Health problem/issue
1	Policy engagement framework	2008	Buse et al. (Buse et al. 2008)	Health Financing (Salehi et al. 2021)
2	Problem-driven framework for applied political economy analysis	2013	Daniel Harris (Harris 2014)	Human Resource for Health (Bertone and Witter 2015; Bertone et al. 2018) Health Financing Governance (Ahsan et al. 2024)
3	The Political Economy of Health Financing Reform Framework	2019	Paola Abril Campos and Michael R. Reich (Campos and Reich 2019)	Health Financing (Croke et al. 2019; Sparkes et al. 2019; Nannini et al. 2021; Nonvignon et al. 2023) Regulatory policies (Thow et al. 2021) Health Reform (Fox and Choi 2019)
4	Institutional political economy approach	2014	David Hudson and Adrian Leftwich (Hudson 2014)	Human Resources for Health (Fieno et al. 2016)
5	Health system governance framework	2009	Siddiqi et al. (Siddiqi et al. 2009)	Governance (Kuwawenaruwa et al. 2023; Rodríguez et al. 2023; Tsofa et al. 2023)
6	‘How to’ notes on Political Economy Analysis prepared by the UK’s Department for International Development	2009	UK’s Department for International Development (DFID) (DFID 2009)	Health Financing (Witter et al. 2019, Hipgrave et al. 2019)
7	HOW-TO NOTES Political Economy Assessments at Sector and Project Levels	2011	World Bank (Poole 2011)	Health Financing (Hipgrave et al. 2019)
8	Problem-driven GPE analysis	2009	Fritz et al. (Fritz et al. 2009)	Health Financing (Hipgrave et al. 2019)
9	Accountability framework	2003	World Bank (Agnès 2004)	Governance (Bukenya and Golooba-Mutebi 2020)
10	Walt and Gilson's policy triangle conceptual framework	1994	Gill Walt and Lucy Gilson (Walt and Gilson 1994)	Health Reform (Widdig et al. 2022) Governance (Bigirinama et al. 2024)
11	Shiffman’s framework for global health policy	2007	Jeremy Shiff man and Stephanie Smith (Shiffman and Smith 2007)	Regulatory Policies (Mounsey et al. 2022)
12	Kingdon’s ‘multiple streams’ theory	2003	John W Kingdon (Kingdon 1995)	Regulatory Policies (Mounsey et al. 2022)
13	Framework for organizing political analyses of health reform in developing countries	2015	Ashley M. Fox and Michael R. Reich (Campos and Reich 2019)	Health Financing (Nannini et al. 2021)
14	The Policy Circle framework	2004	Hardee et al. (Hardee et al. 2004)	Health Reform (Gómez-Dantés et al. 2015)
15	The Andersen Behaviour Model	1968	Andersen (Andersen 1968)	Health Reform (Gómez-Dantés et al. 2015)
16	Stakeholder analysis	2000	Varvasovszky (Varvasovszky and Brugha 2000)	Health Reform (Gómez-Dantés et al. 2015) Health Financing (Croke et al. 2019)
17	Political settlement theory	2010	Mushtaq H. Khan (Khan 2010)	Governance (Bukenya and Golooba-Mutebi 2020)
18	Veto gates theory	1992	Ellen M. Immergut (Immergut 1992)	Health Financing (Croke et al. 2019)
19	Historical institutionalist theory	1996	Paul Pierson (Pierson and Skocpol 2002)	Health Financing (Croke et al. 2019)
20	Applied political analysis for health policy reform	1996	Michael R. Reich (Reich 1996)	Health Financing (Croke et al. 2019)
**21**	Macro-level Model of Employment Relations	2010	Muntaner et al. (Muntaner et al. 2010)	Health Equity (Fujishiro et al. 2021)
22	Social basis of disparities in health	2001	Diderichsen et al. (Diderichsen et al. 2001)	Health Equity (Fujishiro et al. 2021)
23	Interest group theory	2003	Rajan RG and Zingales (Rajan and Zingales 2003)	Health Financing (Croke et al. 2019)
24	Path dependence	2004 2012	Paul Pierson (Pierson 2004) Haeder (Haeder 2012)	Health Reform (Tangcharoensathien et al. 2019)
25	Median voter theory	1948	Black D (Black 1948)	Voter preferences and referendum outcomes (dental policy) (Norris 2025)
26	Political Settlement Analysis	1995	Khan M H (Khan 2010)	Health reform (Croke and Ogbuoji 2024)
27	3Is + Power Framework	2014	Pettit and Mejía Acosta (Pettit 2013)	Governance (Sing et al. 2025)
28	Four I’s Framework	2015	Fox & Reich (Fox and Reich 2015)	Governance (Sing et al. 2025)
29	Integrated Political Economy Framework	2020	Rizvi et al. (Rizvi et al. 2020)	Governance, Health Financing (Chukwuma and Obi 2025)
30	Public Choice Theory	1962	James M. Buchanan, Gordon Tullock (Buchanan and Tullock 1965)	Inefficiencies in public health spending; misallocation of health resources due to political incentives (Norris 2025)
31	Rent-Seeking Theory	1974	Gordon Tullock, Anne Krueger (Tullock 2008)	Pharmaceutical lobbying leading to inflated drug prices; regulatory capture in health policy (Norris 2025)

In more politically competitive settings, as illustrated by Norris (2025), Median Voter Theory (Black 1948), Public Choice Theory (Buchanan and Tullock 1965), and Rent-Seeking Theory (Krueger 2008, Tullock 2008) were used to explain how stakeholder interests and voter preferences shaped the outcome of Washington’s dental policy referendum. These theories interpret policymaking as the outcome of individual or group self-interest operating within institutional constraints—where actors seek to maximize political or economic gains (rent-seeking) and align with the median voter to secure policy support (public choice). Such perspectives—though grounded in political science—are often integrated into PEA when analysing direct democracy, electoral accountability, or resistance to regulatory reforms. Some studies did not explicitly mention a specific framework (Barraclough and Morrow 2017).

These findings demonstrate that no single framework is sufficient to capture the multidimensional nature of the political economy in health. Instead, studies often adapted and combined multiple frameworks—drawing from diverse disciplines—to address the complex nature of health systems research questions. This flexible, hybrid approach enabled researchers to account for both structural and agency-driven dynamics and to better contextualize their findings across different political and institutional settings. While a single comprehensive framework might offer conceptual coherence, the diversity of political and institutional contexts suggests that adaptable, problem-driven frameworks are more desirable for capturing the contextual specificity of health policy processes.

#### The rationale for using PEA

The adoption of PEA across the 28 studies was driven by diverse rationales aimed at comprehensively understanding and navigating complex health issues (See Table 2). PEA served as a crucial lens for understanding the influence of diverse actors and organizations on policy adoption and implementation (Bertone and Witter 2015, Salehi et al. 2021), as well as evaluating the potential impact of reform experiences (Sparkes et al. 2019, Tangcharoensathien et al. 2019, Nannini et al. 2021). By emphasizing the importance of political economy factors, these studies aimed to illuminate critical policy trajectories (Sparkes et al. 2019 ), shedding light on the dynamics of policy-making, leadership accountability, and the prioritization of development objectives (Fieno et al. 2016). PEA also played a pivotal role in understanding the intricacies of health workforce recruitment (Bertone et al. 2018), sub-national health management (Rodríguez et al. 2023, Tsofa et al. 2023, Ahsan et al. 2024) and allocation of resources for maternal, new-born, and child health (Hipgrave et al. 2019, Bigirinama et al. 2024). Similarly, it also illustrated how political turnover, institutional fragmentation, and weak enforcement hampered sustained implementation (Croke and Ogbuoji 2024). It also analysed the dynamics influencing HMIS investment and strengthening (Kuwawenaruwa et al. 2023). It served as a valuable tool for examining the drivers and constraints within various health policy domains (Mounsey et al. 2022), including the adoption of social protection policies, the implications of health financing reforms (Gómez-Dantés et al. 2015), the historical nexus between ethnicity and tobacco control (Barraclough and Morrow 2017) and the persistent reliance of donors and governments on external funding for traditional vaccines (Nonvignon et al. 2023). In high-income settings, PEA has also helped assess barriers to implementing legislative responses to unhealthy food marketing (Sing et al. 2025). Their analysis revealed how neoliberal ideologies, institutional norms, and commercial interests limited the scope of regulation despite strong public health mandates.

#### Theoretical/conceptual backing

The theoretical foundations of PEA in these studies span economics, political science, and sociology. Focused on contextual factors, power dynamics, and path dependency (Salehi et al. 2021), different theories/concepts explain how historical legacies and socio-economic conditions mould policy trajectories (Table 2). Economic theories, such as Agency Theory (Kiser 1999) and Stewardship Theory (Perrow 1986), focus on the motivations of key actors, bridging economic thinking with political processes. Political theories, encompassing Interest Group Politics, Bureaucratic Politics, Budget Politics, Leadership Politics, Beneficiary Politics and political settlement theory, highlight the process of political interactions shaping policies (Campos and Reich 2019). The interplay between economic and political realms, with concepts like Structural Diagnosis, Agency Diagnosis (Tsofa et al. 2023), ‘Theory of Change,’ and ‘Drivers of Change’ (Hipgrave et al. 2019) explains policy cycle complexities and driving factors. Frameworks by Shiffman and Smith (2007) and Kingdon (1995), along with concepts like Interest Group Theory (Rajan and Zingales 2003), Veto Gates Theory (Immergut 1992), Historical Institutionalism (Pierson 1993), and Stakeholder Theory (Croke et al. 2019, Hipgrave et al. 2019, Nannini et al. 2021), deepen understanding of policy evolution, negotiation dynamics, and implementation outcomes. The Theory of Discursive Institutionalism (Schmidt 2008) was utilized to explore socio-cultural dimensions influencing policy decisions. Some studies explicitly detail theoretical foundations (Bertone and Witter 2015, Croke et al. 2019, Hipgrave et al. 2019, Bukenya and Golooba-Mutebi 2020, Fujishiro et al. 2021, Thow et al. 2021, Mounsey et al. 2022), while others rely on path dependency (Croke et al. 2019, Tangcharoensathien et al. 2019, Salehi et al. 2021) to explore historical trajectories shaping policy outcomes.

These findings suggest that frameworks are often used in combination rather than in isolation to capture the multi-level, dynamic nature of political economy in health. Studies are increasingly adapting or blending elements from multiple frameworks to better respond to complex research questions in health systems, reflecting a shift towards flexible, context-responsive applications of PEA.

### Review question 3: What health problems are addressed, and how is political economy analysis applied in understanding and addressing them?

A wide range of issues was examined using PEA (Table 1), spanning financing, governance, human resources for health (HRH), service delivery/HMIS, and regulation of harmful commodities. Across domains, application of PEA followed recurring analytical moves: (i) actor/coalition mapping and incentive diagnosis; (ii) institutional analysis of formal and informal rules, legacies, and veto points; (iii) ideas/discourse analysis to understand frames and legitimacy; and (iv) implementation politics—how capacity, resources, and power asymmetries shape what happens on the ground. Below we summarize how these moves were executed within each domain.

#### Health financing

Several studies applied PEA to analyse health financing reforms, including performance-based financing in Afghanistan (Salehi et al. 2021), results-based financing in Zimbabwe (Witter et al. 2019), maternal and child health financing in Asia (Hipgrave et al. 2019), and insurance debates in Uganda (Nannini et al. 2021). These analyses traced how global policy contexts, institutional legacies, and bargaining among political and technical actors influenced financing trajectories. For example, Sparkes et al. (2019) showed that health financing reform was inherently political, with technical design and political feasibility intertwined, while (Croke et al. 2019) demonstrated how veto points and interest group mobilization persistently blocked reform in Malaysia. PEA thus moved beyond fiscal constraints to expose the political conditions that enabled or obstructed reform adoption and sustainability.

#### Governance

Governance was the most extensively studied domain, where PEA illuminated how decentralization, elite bargains, and donor influence shaped health sector reforms. In Kenya, actor and institutional analyses were used to demonstrate how local political interests mediated the interpretation and implementation of national agendas in county-level decision-making. In Mexico, Gómez-Dantés et al. (2015) highlighted how powerful actors could leverage favourable contexts to advance social protection policies without broad citizen mobilization. Similarly, Bukenya and Golooba-Mutebi (2020) that informal institutional contexts explained sub-national differences in maternal mortality in Uganda, while Namakula et al. (2025) revealed persistent bureaucratic barriers and weak accountability constraining subnational management. These applications emphasize that governance reforms cannot be understood as neutral technical processes but must be situated in the interplay of actors, institutions, and ideas.

#### Human resources for health (HRH)

PEA was widely applied to HRH issues, particularly in fragile and post-conflict contexts such as Sierra Leone, Timor-Leste, and Ethiopia (Bertone and Witter 2015, Fieno et al. 2016, Bertone et al. 2018). Analyses showed how recruitment practices, incentive structures, and workforce deployment were embedded in political settlements, patronage systems, and competing agendas. For instance, Bertone and Witter (2015) traced how informal practices and actor incentives influenced HRH reforms, while Fieno et al. (2016) argued that political will and strong state capacity were essential to overcoming institutional hurdles in Ethiopia. By linking HRH reforms to broader political structures, these studies illustrated how frontline implementation outcomes were shaped by bargaining, power relations, and institutional weakness.

#### Service delivery and health information systems

Fewer studies examined service delivery and health information systems, but PEA offered valuable insights. In South Sudan, Widdig et al. (2022) used PEA to show how donor dominance in pooled funding arrangements limited national government ownership, constraining service delivery priority-setting. Kuwawenaruwa et al. (2023) applied PEA to health management information systems in Tanzania, revealing how technical reforms were shaped by political drivers such as government–stakeholder relationships, long-term partnerships, and resource allocation battles. These applications demonstrate how service delivery and information systems are deeply political, with authority and power asymmetries shaping outcomes as much as technical design.

#### Regulation and public health policy

Emerging applications of PEA focused on regulatory arenas such as sugar-sweetened beverage taxation (Mounsey et al. 2022), tobacco control (Barraclough and Morrow 2017), restrictions on unhealthy food and breastmilk substitute marketing (Thow et al. 2021), and cross-country analyses of food marketing legislation (Sing et al. 2025). These studies applied actor mapping, discourse analysis, and power frameworks to show how corporate lobbying, framing strategies, and policy entrepreneurs influenced the trajectory of regulation. For example, Sing et al. (2025) demonstrated how neoliberal institutional norms constrained action, while discursive framing around child protection created openings for regulation. Such applications illustrate the adaptability of PEA to contested policy spaces where health and commercial interests collide.

#### Cross-cutting synthesis

Taken together, the findings show that PEA has been applied across all health system domains, but with particular concentration in governance and HRH. Across cases, PEA was not confined to describing reform contexts but served as a lens to analyse how coalitions of actors, institutional legacies, and political incentives shaped both design and implementation. A cross-cutting insight is that PEA consistently revealed the ‘politics of implementation,’ explaining why similar reforms succeeded in some contexts but faltered in others. The applications in emerging areas such as regulation and information systems show the growing adaptability of PEA to novel public health challenges. These insights underline the value of PEA as a methodological approach that connects high-level reforms to the micro-politics of decision-making and frontline implementation.

### Review question 4. What are the reported outcomes (and measures) used in the political analysis of health?

#### Outcomes identified

The political analysis of health yielded key outcomes across various thematic categories (Table 1). Studies investigated contextual factors, spanning from the global policy level (Tangcharoensathien et al. 2019) to national or state-level, with a focus on structural features such as institutional capacity (Gómez-Dantés et al. 2015, Bertone et al. 2018, Hipgrave et al. 2019, Fujishiro et al. 2021, Thow et al. 2021, Mounsey et al. 2022, Tsofa et al. 2023). Another significant outcome revolves around stakeholder analysis, which is bifurcated into the roles of actors (Gómez-Dantés et al. 2015, Fieno et al. 2016, Barraclough and Morrow 2017, Bertone et al. 2018, Fox and Choi 2019, Sparkes et al. 2019, Tangcharoensathien et al. 2019, Thow et al. 2021, Mounsey et al. 2022, Nonvignon et al. 2023) and their power dynamics (Gómez-Dantés et al. 2015, Barraclough and Morrow 2017, Tangcharoensathien et al. 2019, Bukenya and Golooba-Mutebi 2020, Fujishiro et al. 2021) in shaping health outcomes. The political process, encompassing elements like political will, opportunities, and commitment to the health issue, is identified (Gómez-Dantés et al. 2015, Bertone et al. 2018, Tangcharoensathien et al. 2019, Fujishiro et al. 2021, Nannini et al. 2021, Salehi et al. 2021, Kuwawenaruwa et al. 2023, Nonvignon et al. 2023, Rodríguez et al. 2023, Tsofa et al. 2023). Socio-cultural influences, including factors such as ethnicity, are also examined for their impact on health policies (Croke et al. 2019). Priority setting (Fieno et al. 2016, Bukenya and Golooba-Mutebi 2020, Thow et al. 2021) and planning and budgeting (Fieno et al. 2016, Tsofa et al. 2023, Ahsan et al. 2024) were outcomes of the decision-making processes within the policy formulation arena. Inter-sectoral coordination (Thow et al. 2021, Mounsey et al. 2022) emerges as a crucial aspect for the successful implementation of policies, alongside insights gleaned from implementation experiences (Tangcharoensathien et al. 2019). In addition to these, the adoption or blocking of reforms, voting behaviour patterns (e.g. in direct democracy settings), (Croke and Ogbuoji 2024, Norris 2025) and stakeholder alignment with policy outcomes are key observable indicators (Sing et al. 2025). Implementation-specific outcomes include funding disbursement patterns, institutional responsiveness, and programme continuity. Further, process indicators (e.g. legislative progression, coalition-building success) and qualitative measures (e.g. shifts in discourse, legitimacy gains, and framing strategies) (Chukwuma and Obi 2025, Sing et al. 2025) are used to evaluate the political feasibility and sustainability of health interventions. These diverse outcome measures offer nuanced insights into how political and institutional arrangements shape health system reforms and public health outcomes (Table 1).

These findings demonstrate that outcomes in political analysis of health are both diverse and multi-layered, encompassing structural, processual, behavioural, and implementation domains. A wide range of outcome measures—quantitative (e.g. funding disbursement, institutional performance) and qualitative (e.g. discourse shifts, stakeholder power dynamics)—were employed to trace how political and institutional forces influence health system change. This diversity underscores the complexity of measuring ‘success’ or ‘impact’ in political economy studies and the need for mixed-method approaches to fully capture them.

## Discussion

By clarifying definitions, consolidating available frameworks, and mapping their applications across diverse health system issues, this scoping review provides a structured foundation for both researchers and policy actors. Such synthesis is essential for moving PEA from abstract theory into a practical tool that can inform policy planning, guide implementation processes, and support the evaluation of reforms in complex systems. Throughout this scoping review, the term ‘health outcomes’ refer to population- or system-level effects—such as changes in service coverage, institutional responsiveness, or equity in access—rather than individual clinical outcomes. The terms health issues and health problems are used to denote health system or policy challenges, including financing, governance, human resources for health, and service delivery. These distinctions are important because political economy analysis, as applied in the reviewed studies, primarily investigates how political and institutional dynamics shape the functioning and performance of health systems, rather than direct biomedical or epidemiological outcomes.

A key finding of this scoping review is the lack of uniformity in defining PEA. While some studies adopted explicit definitions drawing on established sources (Fieno et al. 2016, Fox and Choi 2019, Salehi et al. 2021), others relied on context-specific interpretations or avoided definition altogether. This inconsistency limits the comparability of results across studies and undermines efforts to build cumulative knowledge. This lack of definitional consensus also influences how PEA is applied in practice, with researchers drawing on a broad range of frameworks to translate political economy concepts into empirical analysis.

Across five decades of research, frameworks such as the Andersen Behaviour Model (Andersen 1968) and Campos & Reich’s Political Economy of Health Financing Reform (Campos and Reich 2019) illustrate the evolution of PEA thought—from its conceptual origins to structured applications. Their diversity highlights the complexity of health systems and underscores the need for adaptable, multifaceted approaches. For instance, the ‘Policy Engagement Framework’ (Buse et al. 2008) is often recommended for addressing financing challenges, the ‘Health System Governance Framework’ (Siddiqi et al. 2009) for governance, and Harris’s ‘Problem-Driven Political Economy Analysis’ (Harris 2014) for both human resources and financing, demonstrating its versatility (Harris 2014).

PEA in this scoping review drew on diverse theoretical foundations spanning economics, political science, and sociology. Economic theories such as Agency and Stewardship (Kiser 1999) were combined with political theories including Interest Group (Rajan and Zingales 2003), Bureaucratic Politics (Sparkes et al. 2019), Political Settlement (Khan 2010), and Historical Institutionalism (Pierson and Skocpol 2002), offering nuanced insights into how interests, institutions, and legacies shape policy trajectories. Applications illustrate this diversity: in Nigeria, (Chukwuma and Obi 2025) used the Rizvi et al. 2020 framework—an adaptation of Fox and Reich 2015 ‘four Is’—to show how entrenched ideologies and interest groups constrained universal coverage reforms, while Croke and Ogbuoji 2024 applied Grindle and Thomas 1991's Politics of Policy Reform framework to highlight how reformist elites enabled adoption but clientelist settlements undermined implementation. More recent analyses explicitly integrate power perspectives: Harris (2014) employed Pettit’s power analysis (2013), Luke’s’ three dimensions (Lorenzi 2006), and Gaventa’s Power Cube (2006) to demonstrate how neoliberal ideologies and discursive strategies shaped food marketing legislation outcomes across Chile, Canada, and the UK. This plurality of approaches reflects the inherently interdisciplinary nature of political economy in health, which draws insights from economics, political science, and sociology to examine how power and institutions shape policy processes. As Duncan Foley (1999) incisively observed, political economy reveals ‘partial truths’ rather than a single, unified theory—each framework offering a different lens to interrogate the relationship between politics and economics. This insight is particularly relevant to the studies reviewed here, where frameworks were selectively adapted to suit context-specific policy questions rather than applied uniformly. Recognizing this diversity as methodological pluralism, rather than inconsistency, highlights how political economy can accommodate both macro-level critique and meso- or micro-level applications in health research.

Beyond domain-specific findings, a cross-cutting application pattern emerged across studies. Effective PEAs were explicit about the problem level (macro, meso, or micro), mapped actor configurations and incentives, specified institutions (formal or informal rules, legacies, and veto points), identified ideas and frames shaping discourse, and analysed implementation politics such as capacity, resources, and power asymmetries (Harris 2014, Fox and Reich 2015, Campos and Reich 2019) When these analytical components were linked sequentially to policy stages, PEA produced richer explanatory insights. Based on this synthesis, PEA applications can be viewed procedurally:

1. define the problem and level;
2. map actors and coalitions;
3. specify institutional context;
4. identify dominant ideas and frames;
5. analyse implementation politics; and
6. infer implications for feasible reform options.

Outcomes generated from these steps—such as reform adoption or blockage, resource allocation shifts, or discourse change—were reflected under RQ4. This structured sequence bridges descriptive mapping and practical application, offering a replicable heuristic for future researchers.

The procedural logic of PEA, as outlined above, can also be interpreted across macro, meso, and micro levels, highlighting how analysis moves from broad structural forces to local implementation realities (Harris 2014, Fox and Reich 2015, Campos and Reich 2019). At the macro level, researchers examine overarching political and economic structures, historical legacies, and dominant ideologies that shape health systems and constrain reform options (Harvey 2021, Lynch 2023). The meso level focuses on networks and institutions—governments, bureaucracies, donors, and professional groups—whose interactions mediate these structural forces and influence which policies advance (Pierson 1993, Varvasovszky and Brugha 2000, Khan 2010). The micro level centres on the ‘real politics of reform’ at the point of implementation, where local managers, health workers, and communities negotiate authority, resources, and incentives within institutional and political constraints (Booth 2016). Viewed through this lens, PEA bridges theory and practice: it links structural critique with practical insights, explaining not only why reforms succeed or fail at the national level but also how they unfold in day-to-day operations. This multilevel interpretation complements the procedural steps outlined above and operationalizes Foley’s idea of ‘partial truths’, acknowledging that each analytical perspective provides only a partial view, yet collectively they offer a richer understanding of policy dynamics (Foley 1999, Schmidt 2008).

Cross-cutting patterns emerged when applications were organised using WHO building blocks. Governance and human resources for health were most frequently analysed, underscoring areas where politics and power asymmetries are most visible (Fieno et al. 2016, Bertone et al. 2018, Tsofa et al. 2023). By contrast, service delivery and health information systems remain underexplored, despite being equally shaped by political incentives, institutional path-dependencies, and resource contests (Kuwawenaruwa et al. 2023). This uneven application suggests that PEA has primarily been deployed as a diagnostic tool in contested domains such as health financing and workforce management, but its potential to inform everyday delivery and monitoring functions has yet to be realized.

Given the heterogeneity of included studies, this scoping review offers a descriptive mapping rather than a comparative synthesis. Future analyses should systematically compare frameworks to evaluate their analytical depth, practical utility, and contextual adaptability.

The strength of this scoping review lies in its pioneering mapping of the literature on political economy analysis in health. The inclusion and exclusion criteria were designed to be broad enough to capture multiple health domains yet narrow enough to focus on structured applications of PEA. We acknowledge that defining ‘structured PEA’ may have excluded conceptual or implicitly political analyses—an inherent limitation reflecting both definitional ambiguity and the boundaries of scoping methodologies. Future reviews could expand to include conceptual approaches that enrich theoretical understanding.

Drawing on these insights, particularly the procedural logic above, we outline four key steps for researchers new to PEA to strengthen study design and execution:

1. Define PEA explicitly in your context. Because definitions vary across the literature, it is essential make your definition explicit and then align the working definition with the research objectives and the policy level at which one is working (e.g. national or sub-national).
2. Select an appropriate theoretical or conceptual framework early or a combination of these frame works. Frameworks such as agency theory, stakeholder theory, or discursive institutionalism can help structure the analysis and clarify relationships between actors, institutions, and outcomes.
3. Draw on existing studies to refine the focus. Reviewing how similar issues have been studied using PEA can sharpen your research scope and ensure methodological feasibility.
4. Choose outcomes that capture both political and policy dimensions. This should include not only policy or implementation changes but also ‘softer’ outcomes such as actor alignment, political commitment, and discourse shifts.

## Conclusion

This scoping review demonstrates that while PEA is increasingly applied in health, it remains methodologically fragmented and unevenly distributed across system domains. By synthesizing definitions, frameworks, and applications, the scoping review provides a baseline reference to support more systematic use of PEA in health research and policy. More explicit methodological guidance and greater attention to implementation politics will enable PEA to realize its potential as a practical tool for strengthening health policy and planning in complex contexts.

## Supplementary Material

czaf096_Supplementary_Data

## Data Availability

The data used in this scoping review were derived from publicly accessible sources, including publications and reports. The complete list of data used in this review is available within the article or as supplementary material. Researchers seeking access to the primary data used in the included studies should consult the original publications and contact the corresponding authors or institutions for any specific data requests.

## Reflexivity statement

This review was conceived in response to a practical challenge we encountered while initiating a political economy analysis in the field of oral health, where we noted a lack of methodological guidance. Our collective background shaped the approach and conduct of this review. The author team comprises eight researchers with academic backgrounds in dentistry, public health, health economics, and nursing. Collectively, we bring expertise in health systems, health policy, health economics, and the social determinants of health. Our interdisciplinary composition enabled us to critically engage with the literature on political economy in health and to interpret findings through complementary lenses. Our shared motivation stems from a commitment to advancing oral health research by grounding it in broader political and economic contexts. This reflexive awareness informed both the design and interpretation of this scoping review, particularly in ensuring relevance to under-researched areas such as oral health.
