# Supplementary material for: Political economy analysis of health: a scoping review of concepts, definitions, frameworks, outcomes, and applications
Source: Health Policy Plan. 2026 Jun 29;41(Suppl 1):i91–i110. doi: 10.1093/heapol/czaf096 (PMC13311670; doi:10.1093/heapol/czaf096)

**Supplementary Figure 1. Distribution of included studies by year of publication.**

**Supplementary Figure 2. Global Distribution of Studies in the Scoping Review**


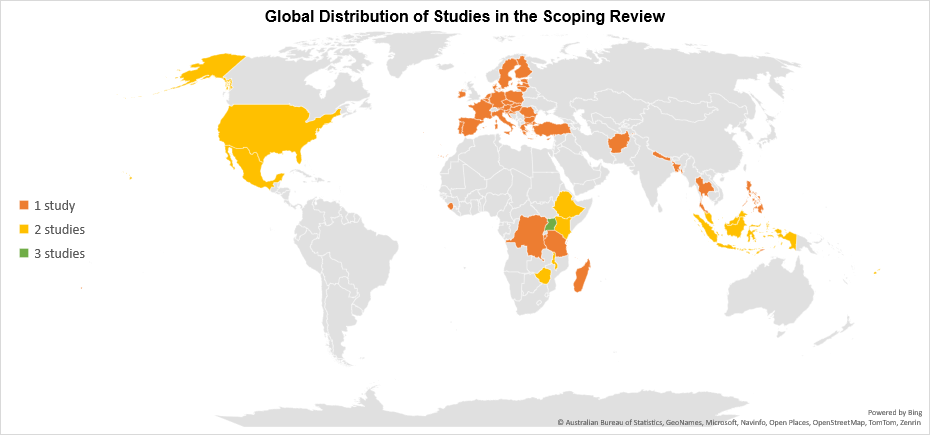


**Supplementary Figure 3: Word cloud derived from definitions of political economy of health**


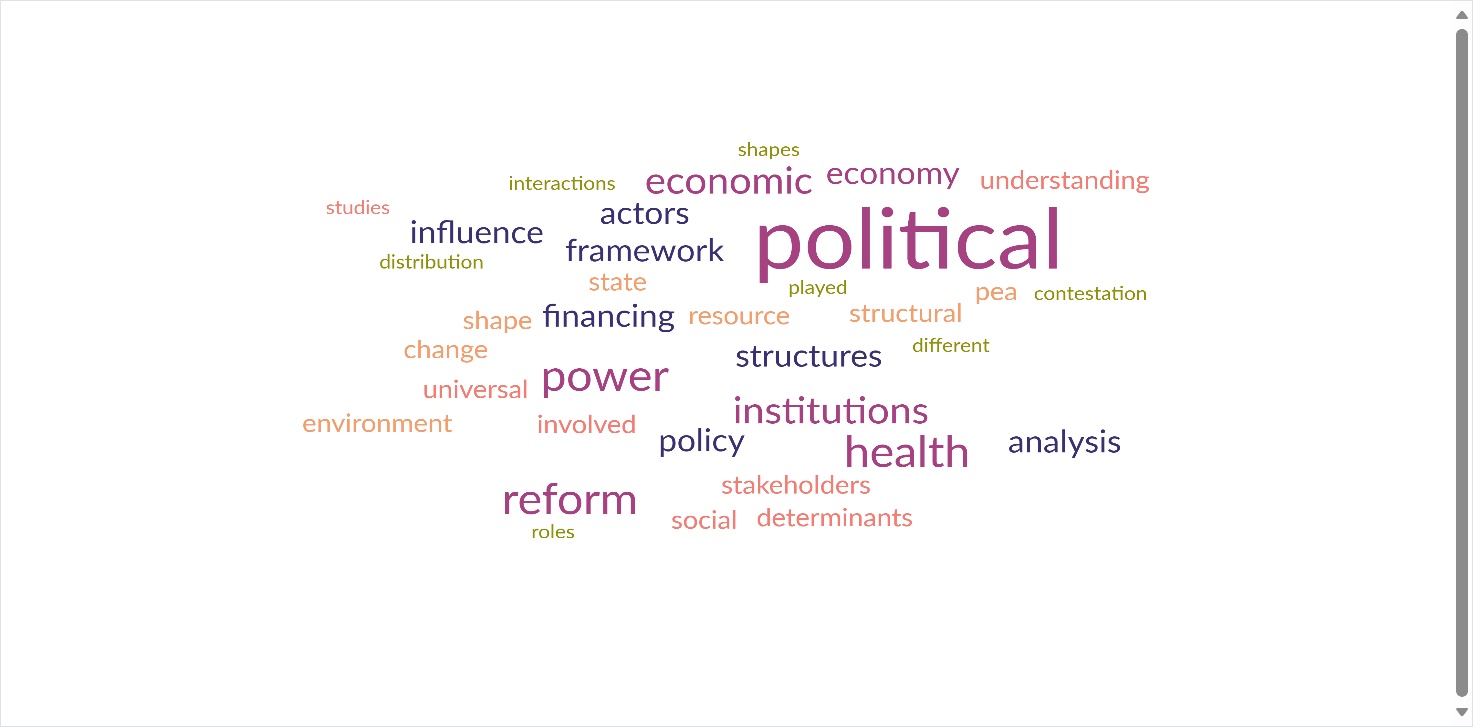

Supplement: czaf096_Supplementary_Data [file czaf096_supplementary_data.zip › Supplementary Figures 1.docx]
