# Supplementary material for: Political economy analysis of health: a scoping review of concepts, definitions, frameworks, outcomes, and applications
Source: Health Policy Plan. 2026 Jun 29;41(Suppl 1):i91–i110. doi: 10.1093/heapol/czaf096 (PMC13311670; doi:10.1093/heapol/czaf096)
Supplement: czaf096_Supplementary_Data [file czaf096_supplementary_data.zip › Supplementary_Table_2 Search strategy.docx]

**Supplementary Table 2: Search strategy**

**MEDLINE (OVID)**

SEARCH DATE: 27-06-2023, Updated 31-05-2024, Updated 05-05-2025

| **Search Query** | **Records** | **Retrieved** |
| --- | --- | --- |
| #1 | "political economy".mp. | 1372 |
| #2 | "political systems".mp. or exp Political Systems/ | 12690 |
| #3 | "health politics".mp. | 455 |
| #4 | "power relationships".mp. | 480 |
| #5 | 1 or 2 or 3 or 4 | 14915 |
| #6 | "financing, government".mp. or exp Financing, Government/ | 114530 |
| #7 | "Government Financing".mp. | 102 |
| #8 | "transnational corporations".mp. | 144 |
| #9 | "welfare states".mp. | 455 |
| #10 | "Healthcare Financing".mp. or exp Healthcare Financing/ | 1801 |
| #11 | "health system".mp. | 57400 |
| #12 | "Health Care Reform".mp. or exp Health Care Reform/ | 38210 |
| #13 | "Health Policy".mp. or exp Health Policy/ | 159000 |
| #14 | exp Public Policy/ or "Public Policy".mp. | 181500 |
| #15 | "Social Determinants of Health".mp. or exp "Social Determinants of Health"/ | 16220 |
| #16 | "Universal Health Care".mp. or exp Universal Health Care/ | 1603 |
| #17 | "Primary Health Care".mp. or exp Primary Health Care/ | 213200 |
| #18 | 6 or 7 or 8 or 9 or 10 or 11 or 12 or 13 or 14 or 15 or 16 or 17 | 571145 |
| #19 | (#5) AND (#18) | 2475 |

**SCOPUS**

SEARCH DATE: 27-06-2023, Updated 31-05-2024, Updated 05-05-2025

| 1 | ( TITLE-ABS-KEY ( "political economy" ) OR TITLE-ABS-KEY ( "political system*" ) OR TITLE-ABS-KEY ( "health politics" ) OR TITLE-ABS-KEY ( "power relationships" ) ) | 89,380 |
| --- | --- | --- |
| 2 | ( TITLE-ABS-KEY ( "financing, government" ) OR TITLE-ABS-KEY ( "Government Financing" ) OR TITLE-ABS-KEY ( "transnational corporations" ) OR TITLE-ABS-KEY ( "welfare states" ) OR TITLE-ABS-KEY ( "Healthcare Financing" ) OR TITLE-ABS-KEY ( "Healthcare Financing" ) OR TITLE-ABS-KEY ( "health system" ) OR TITLE-ABS-KEY ( "Health Care Reform" ) OR TITLE-ABS-KEY ( "Health Policy" ) OR TITLE-ABS-KEY ( "Public Policy" ) OR TITLE-ABS-KEY ( "Social Determinants of Health" ) OR TITLE-ABS-KEY ( "Universal Health Care" ) OR TITLE-ABS-KEY ( uhc ) OR TITLE-ABS-KEY ( "Primary Health Care" ) ) | 5,86,125 |
| 3 | ( ( TITLE-ABS-KEY ( "political economy" ) OR TITLE-ABS-KEY ( "political system*" ) OR TITLE-ABS-KEY ( "health politics" ) OR TITLE-ABS-KEY ( "power relationships" ) ) ) AND ( ( TITLE-ABS-KEY ( "financing, government" ) OR TITLE-ABS-KEY ( "Government Financing" ) OR TITLE-ABS-KEY ( "transnational corporations" ) OR TITLE-ABS-KEY ( "welfare states" ) OR TITLE-ABS-KEY ( "Healthcare Financing" ) OR TITLE-ABS-KEY ( "Healthcare Financing" ) OR TITLE-ABS-KEY ( "health system" ) OR TITLE-ABS-KEY ( "Health Care Reform" ) OR TITLE-ABS-KEY ( "Health Policy" ) OR TITLE-ABS-KEY ( "Public Policy" ) OR TITLE-ABS-KEY ( "Social Determinants of Health" ) OR TITLE-ABS-KEY ( "Universal Health Care" ) OR TITLE-ABS-KEY ( uhc ) OR TITLE-ABS-KEY ( "Primary Health Care" ) ) ) | 6,190 |

**COCHRANE CENTRAL**

SEARCH DATE: 27-06-2023, Updated 31-05-2024, Updated 05-05-2025

| #1 | political economy | 44 |
| --- | --- | --- |
| #2 | political systems | 221 |
| #3 | health politics | 147 |
| #4 | power relationships | 1315 |
| #5 | #1 or #2 or #3 or #4 | 1616 |
| #6 | financing, government | 189 |
| #7 | transnational corporations | 1 |
| #8 | welfare states | 739 |
| #9 | Healthcare Financing | 196 |
| #10 | health system | 59719 |
| #11 | Health Care Reform | 327 |
| #12 | Health Policy | 12389 |
| #13 | Public Policy | 4954 |
| #14 | Social Determinants of Health | 1826 |
| #15 | Universal Health Care | 1847 |
| #16 | Primary Health Care | 77216 |
| #17 | #6 or #7 or #8 or #9 or #10 or #11 or #12 or #13 or #14 or #15 or #16 | 126624 |
| #18 | #5 and #17 | 965 |

**CINHAL (EBSCO)**

SEARCH DATE: 27-06-2023, Updated 31-05-2024, Updated 05-05-2025

| S1 | TX "political economy" OR TX "political system*" OR MH "political systems" OR TX "health politics" OR TX "power relationships" | 9,120 |
| --- | --- | --- |
| S2 | MH "financing, government" OR TX "Government Financing" OR TX "transnational corporations" OR TX "welfare states" OR MH "Healthcare Financing" OR TX "Healthcare Financing" OR TX "health system" OR MH "Health Care Reform" OR TX "Health Care Reform" OR MH "Health Policy" OR TX "Health Policy" | 2,03,000 |
| S3 | TX "Public Policy" OR MH "Public Policy" OR TX "Social Determinants of Health" OR MH "Social Determinants of Health" OR MH "Universal Health Care" OR TX "Universal Health Care" OR TX UHC OR MH "Primary Health Care" OR TX "Primary Health Care" | 1,74,000 |
| S4 | TX "Public Policy" OR MH "Public Policy" OR TX "Social Determinants of Health" OR MH "Social Determinants of Health" OR MH "Universal Health Care" OR TX "Universal Health Care" OR TX UHC OR MH "Primary Health Care" OR TX "Primary Health Care" | 3,39,200 |
| S5 | S1 AND S4 | 3,285 |

**JBI EVIDENCE SYNTHESIS (OVID)**

SEARCH DATE: 27-06-2023, Updated 31-05-2024, Updated 05-05-2025

| Search Query | Records | Retrieved |
| --- | --- | --- |
| #1 | "political economy".mp. | 1 |
| #2 | "political systems".mp. or exp Political Systems/ | 1 |
| #3 | "health politics".mp. | 0 |
| #4 | "power relationships".mp. | 2 |
| #5 | 1 or 2 or 3 or 4 | 4 |
| #6 | "financing, government".mp. or exp Financing, Government/ | 1 |
| #7 | "Government Financing".mp. | 0 |
| #8 | "transnational corporations".mp. | 0 |
| #9 | "welfare states".mp. | 0 |
| #10 | "Healthcare Financing".mp. or exp Healthcare Financing/ | 0 |
| #11 | "health system".mp. | 138 |
| #12 | "Health Care Reform".mp. or exp Health Care Reform/ | 3 |
| #13 | "Health Policy".mp. or exp Health Policy/ | 216 |
| #14 | exp Public Policy/ or "Public Policy".mp. | 18 |
| #15 | "Social Determinants of Health".mp. or exp "Social Determinants of Health"/ | 34 |
| #16 | "Universal Health Care".mp. or exp Universal Health Care/ | 3 |
| #17 | "Primary Health Care".mp. or exp Primary Health Care/ | 120 |
| #18 | 6 or 7 or 8 or 9 or 10 or 11 or 12 or 13 or 14 or 15 or 16 or 17 | 448 |
| #19 | (#5) AND (#18) | 3 |

**EPISTEMONIKOS**

SEARCH DATE: 27-06-2023, Updated 31-05-2024, Updated 05-05-2025

| 1 | (title:("political economy") OR abstract:("political economy")) OR (title:("political systems") OR abstract:("political systems")) OR (title:("health politics") OR abstract:("health politics")) OR (title:("power relationships") OR abstract:("power relationships")) | 345 |
| --- | --- | --- |
| 2 | (title:((title:("financing, government") OR abstract:("financing, government")) OR (title:("Government Financing") OR abstract:("Government Financing")) OR (title:("transnational corporations") OR abstract:("transnational corporations")) OR (title:("welfare states") OR abstract:("welfare states")) OR (title:("transnational corporations") OR abstract:("transnational corporations")) OR (title:("Healthcare Financing".) OR abstract:("Healthcare Financing".)) OR (title:("health system") OR abstract:("health system")) OR (title:("Health Care Reform") OR abstract:("Health Care Reform"))) OR abstract:((title:("financing, government") OR abstract:("financing, government")) OR (title:("Government Financing") OR abstract:("Government Financing")) OR (title:("transnational corporations") OR abstract:("transnational corporations")) OR (title:("welfare states") OR abstract:("welfare states")) OR (title:("transnational corporations") OR abstract:("transnational corporations")) OR (title:("Healthcare Financing".) OR abstract:("Healthcare Financing".)) OR (title:("health system") OR abstract:("health system")) OR (title:("Health Care Reform") OR abstract:("Health Care Reform")))) | 17,510 |
| 3 | (title:((title:("Health Policy") OR abstract:("Health Policy")) OR (title:("Public Policy") OR abstract:("Public Policy")) OR (title:("Social Determinants of Health") OR abstract:("Social Determinants of Health")) OR (title:("Universal Health Care") OR abstract:("Universal Health Care"))) OR abstract:((title:("Health Policy") OR abstract:("Health Policy")) OR (title:("Public Policy") OR abstract:("Public Policy")) OR (title:("Social Determinants of Health") OR abstract:("Social Determinants of Health")) OR (title:("Universal Health Care") OR abstract:("Universal Health Care")))) | 9,730 |
| 4 | 2 OR 3 | 26,406 |
| 5 | 1 AND 4 | 33 |

**OAISTER**

SEARCH DATE: 27-06-2023, Updated 31-05-2024, Updated 05-05-2025

| 1 | kw:("political economy") OR kw:("political systems") OR kw:("health politics") OR kw:("power relationships") | 51,609 |
| --- | --- | --- |
| 2 | kw:("financing, government") OR kw:("Government Financing") OR kw:("transnational corporations") OR kw:("welfare states") OR kw:("transnational corporations") OR kw:("Healthcare Financing") OR kw:("health system") OR kw:("Health Care Reform") | 61,3054 |
| 3 | kw:("Health Policy") OR kw:("Public Policy") OR kw:("Social Determinants of Health") OR kw:("Universal Health Care") | 1,17,200 |
| 4 | kw:("financing, government") OR kw:("Government Financing") OR kw:("transnational corporations") OR kw:("welfare states") OR kw:("transnational corporations") OR kw:("Healthcare Financing") OR kw:("health system") OR kw:("Health Care Reform") OR kw:("Health Policy") OR kw:("Public Policy") OR kw:("Social Determinants of Health") OR kw:("Universal Health Care") | 1,73,500 |
| 5 | kw:("political economy") OR kw:("political systems") OR kw:("health politics") OR kw:("power relationships") AND kw:("financing, government") OR kw:("Government Financing") OR kw:("transnational corporations") OR kw:("welfare states") OR kw:("transnational corporations") OR kw:("Healthcare Financing") OR kw:("health system") OR kw:("Health Care Reform") OR kw:("Health Policy") OR kw:("Public Policy") OR kw:("Social Determinants of Health") OR kw:("Universal Health Care") AND kw:("financing, government") OR kw:("Government Financing") OR kw:("transnational corporations") OR kw:("welfare states") OR kw:("transnational corporations") OR kw:("Healthcare Financing") OR kw:("health system") OR kw:("Health Care Reform") OR kw:("Health Policy") OR kw:("Public Policy") OR kw:("Social Determinants of Health") OR kw:("Universal Health Care") | 1915 |

**PROQuest Thesis and Dissertations**

SEARCH DATE: 27-06-2023, Updated 31-05-2024, Updated 05-05-2025

| S1 | summary("political economy") OR mainsubject("political systems") OR summary("political systems") OR summary("health politics") OR "power relationships" | 4012 |
| --- | --- | --- |
| S2 | summary("financing, government") OR mainsubject("financing, government") OR summary("Government Financing") OR summary("transnational corporations") OR summary("welfare states") OR "transnational corporations" OR mainsubject("Healthcare Financing") OR summary("health system") OR mainsubject("Health Care Reform") OR summary("Health Care Reform") | 36,761 |
| S3 | mainsubject("Health Policy") OR summary("Health Policy") OR mainsubject("Public Policy") OR summary("Public Policy") OR mainsubject("Social Determinants of Health") OR summary("Social Determinants of Health") OR mainsubject("Universal Health Care") OR summary("Universal Health Care") | 37,700 |
| S4 | [S2] OR [S3] | 71,532 |
| S5 | [S1] AND [S4] | 388 |

**EMBASE**

SEARCH DATE: 24-11-2025 Updated 05-05-2025

| 1 | "political economy".mp. | 1387 |
| --- | --- | --- |
| 2 | "political systems".mp. OR exp "Political Systems"/ | 15391 |
| 3 | "health politics".mp. | 579 |
| 4 | "power relationships".mp. | 541 |
| 5 | 1 OR 2 OR 3 OR 4 | 17645 |
| 6 | "financing, government".mp. OR exp "Financing, Government"/ | 389 |
| 7 | "Government Financing".mp. | 127 |
| 8 | "transnational corporations".mp. | 130 |
| 9 | "welfare states".mp. | 452 |
| 10 | "Healthcare Financing".mp. OR exp "Healthcare Financing"/ | 14621 |
| 11 | "health system".mp. | 88923 |
| 12 | "Health Care Reform".mp. OR exp "Health Care Reform"/ | 238996 |
| 13 | "Health Policy".mp. OR exp "Health Policy"/ | 256117 |
| 14 | exp "Public Policy"/ OR "Public Policy".mp. | 264708 |
| 15 | "Social Determinants of Health".mp. OR exp "Social Determinants of Health"/ | 33491 |
| 16 | "Universal Health Care".mp. OR exp "Universal Health Care"/ | 2594 |
| 17 | "Primary Health Care".mp. OR exp "Primary Health Care"/ | 237931 |
| 18 | 6 OR 7 OR 8 OR 9 OR 10 OR 11 OR 12 OR 13 OR 14 OR 15 OR 16 OR 17 | 621387 |
| 19 | (#5 ) AND (#18 ) | 3904 |

**Google Scholar**

SEARCH DATE: 27-06-2023 Updated 31-05-2024 Updated 05-05-2025

First 10 pages

| 1 | ("political economy" OR "health politics" OR "power relations" OR "political systems") AND ("health policy" OR "health system" OR "universal health coverage" OR "primary health care") | 204 |
| --- | --- | --- |
