# Supplementary material for: Political economy analysis of health: a scoping review of concepts, definitions, frameworks, outcomes, and applications
Source: Health Policy Plan. 2026 Jun 29;41(Suppl 1):i91–i110. doi: 10.1093/heapol/czaf096 (PMC13311670; doi:10.1093/heapol/czaf096)
Supplement: czaf096_Supplementary_Data [file czaf096_supplementary_data.zip › Supplementary_Table_3 List of excluded studies.docx]

**Supplementary Table 2: Studies excluded on full text**

| **S.No** | **Studies excluded** | **Reason for exclusion** |
| --- | --- | --- |
| 1. | Sparkes SP, Campos Rivera PA, Jang H, Marten R, Rajan D, Robb A, Shroff ZC. Normalizing the political economy of improving health. Bull World Health Organ. 2022 Apr 1;100(4):276-280. doi: 10.2471/BLT.21.286629. Epub 2022 Mar 3. PMID: 35386555; PMCID: PMC8958830. | No structured political economy analysis |
| 2. | Jakovljevic, M., Jakab, M., Gerdtham, U., McDaid, D., Ogura, S., Varavikova, E., … Getzen, T. E. (2019). Comparative financing analysis and political economy of noncommunicable diseases. Journal of Medical Economics, 22(8), 722–727. https://doi.org/10.1080/13696998.2019.1600523 | Review Article |
| 3. | Ssennyonjo A. Beyond "Lack of Political Will": Elaborating Political Economy Concepts to Advance "Thinking and Working Politically" Comment on "Health Coverage and Financial Protection in Uganda: A Political Economy Perspective". Int J Health Policy Manag. 2023;12:7297. doi: 10.34172/ijhpm.2022.7297. Epub 2022 May 22. PMID: 35643421; PMCID: PMC10125056. | Commentary |
| 4. | Schrecker T, Birn AE, Aguilera M. How extractive industries affect health: Political economy underpinnings and pathways. Health Place. 2018 Jul;52:135-147.doi: 10.1016/j.healthplace.2018.05.005. Epub 2018 Jun 7. PMID: 29886130. | No structured political economy analysis |
| 5. | Hsiao WC. The political economy of Chinese health reform. Health Economics, Policy and Law. 2007 Jul;2(3):241-9. | Editorial |
| 6. | Loffreda, G., Bello, K., Kiendrébéogo, J.A. et al. Political economy analysis of universal health coverage and health financing reforms in low- and middle-income countries: the role of stakeholder engagement in the research process. Health Res Policy Sys 19, 143 (2021). https://doi.org/10.1186/s12961-021-00788-w | Review article |
| 7. | Mhazo AT, Maponga CC. The political economy of health financing reforms in Zimbabwe: a scoping review. Int J Equity Health. 2022 Mar 27;21(1):42. doi: 10.1186/s12939-022-01646-z. PMID: 35346208; PMCID: PMC8962130. | Review article |
| 8. | McCartney G, Hearty W, Arnot J, Popham F, Cumbers A, McMaster R. Impact of Political Economy on Population Health: A Systematic Review of Reviews. Am J Public Health. 2019 Jun;109(6):e1-e12. doi: 10.2105/AJPH.2019.305001. PMID: 31067117; PMCID: PMC6507992. | Review article |
| 9. | Bagus P, Peña-Ramos JA, Sánchez-Bayón A. COVID-19 and the Political Economy of Mass Hysteria. International Journal of Environmental Research and Public Health. 2021; 18(4):1376. https://doi.org/10.3390/ijerph18041376 | No structured political economy analysis |
| 10. | Ssennyonjo A. Beyond "Lack of Political Will": Elaborating Political Economy Concepts to Advance "Thinking and Working Politically" Comment on "Health Coverage and Financial Protection in Uganda: A Political Economy Perspective". Int J Health Policy Manag. 2023;12:7297. doi: 10.34172/ijhpm.2022.7297. Epub 2022 May 22. PMID: 35643421; PMCID: PMC10125056. | Commentary |
| 11. | Rudiger A. Human Rights and the Political Economy of Universal Health Care: Designing Equitable Financing. Health Hum Rights. 2016 Dec;18(2):67-78. PMID: 28559677; PMCID: PMC5395005. | Commentary |
| 12. | Glasgow, S., & Schrecker, T. (2015). The double burden of neoliberalism? Noncommunicable disease policies and the global political economy of risk. Health & Place, 34, 279–286. doi:10.1016/j.healthplace.2015.06 | No structured political economy analysis |
| 13. | Jakovljevic, M., Liu, Y., Cerda, A., Simonyan, M., Correia, T., Mariita, R. M., … Varjacic, M. (2021). The Global South political economy of health financing and spending landscape – history and presence. Journal of Medical Economics, 24(sup1), 25–33. https://doi.org/10.1080/13696998.2021.2007691 | Review article |
| 14. | Benton, A., & Dionne, K. Y. (2015). International Political Economy and the 2014 West African Ebola Outbreak. African Studies Review, 58(01), 223–236. doi:10.1017/asr.2015.11 | Commentary |
| 15. | Stuckler, D., Feigl, A. B., Basu, S., & McKee, M. (2010, November). The political economy of universal health coverage. In Background paper for the global symposium on health systems research. Geneva: World Health Organization (Vol. 2010). | Symposium Proceedings |
| 16. | Bambra C. Work, worklessness and the political economy of health inequalities. Journal of Epidemiology & Community Health. 2011 Sep 1;65(9):746-50. | Commentary |
| 17. | Szreter S, Woolcock M. Health by association? Social capital, social theory, and the political economy of public health. International journal of epidemiology. 2004 Aug 1;33(4):650-67. | Commentary |
| 18. | Gilson L. Reflections from South Africa on the value and application of a political economy lens for health financing reform. Health Systems & Reform. 2019 Jul 3;5(3):236-43. | Commentary |
| 19. | Morgan LM. Dependency theory in the political economy of health: an anthropological critique. Medical Anthropology Quarterly. 1987 Jun;1(2):131-54. | Commentary |
| 20. | Donahue JM, McGuire MB. The political economy of responsibility in health and illness. Social Science & Medicine. 1995 Jan 1;40(1):47-53. | No structured political economy analysis |
| 21. | Gish O. The political economy of primary care and “health by the people”: an historical exploration. Social Science & Medicine. Part C: Medical Economics. 1979 Dec 1;13(4):203-11. | Review article |
| 22. | Chauhan V, Dumka N, Hannah E, Ahmed T, Kotwal A. Recent initiatives for transforming healthcare in India: A political economy of health framework analysis. Journal of Global Health Economics and Policy. 2022 Apr 25;2:e2022002. | Review article |
| 23. | Gilson L, Russell S, Buse K. The political economy of user fees with targeting: developing equitable health financing policy. Journal of international Development. 1995 May;7(3):369-401. | Review article |
| 24. | Friel S, Jamieson L. Political economy, trade relations and health inequalities: lessons from general health. | No structured political economy analysis |
| 25. | Navarro V, Shi L. The political context of social inequalities and health. International Journal of Health Services. 2001 Jan;31(1):1-21. | No structured political economy analysis |
| 26. | Alford RR. The political economy of health care: Dynamics without change. Politics & society. 1972 Mar;2(2):127-64. | Commentary |
| 27. | Basaza RK, Namyalo PK, Mutatina B. Health Financing Reforms in Uganda: Dispelling the Fears and Misconceptions Related to Introduction of a National Health Insurance Scheme: Comment on" Health Coverage and Financial Protection in Uganda: A Political Economy Perspective". International Journal of Health Policy and Management. 2023;12. | Commentary |
| 28. | Carpenter D. Is health politics different?. Annual Review of Political Science. 2012 Jun 15;15:287-311. | Review Article |
| 29. | Shadlen KC. The political economy of AIDS treatment: intellectual property and the transformation of generic supply. International Studies Quarterly. 2007 Sep 1;51(3):559-81. | Review Article |
| 30. | Boettke P, Powell B. The political economy of the COVID‐19 pandemic. Southern Economic Journal. 2021 Apr;87(4):1090-106. | Symposium proceedings |
| 31. | Bakker I. Social reproduction and the constitution of a gendered political economy. New political economy. 2007 Dec 1;12(4):541-56. | Review Article |
| 32. | Moncrieff J. The Political Economy of the Mental Health System: A Marxist Analysis. Front Sociol. 2022 Jan 17;6:771875. doi: 10.3389/fsoc.2021.771875. PMID: 35242843; PMCID: PMC8886881. | Review Article |
| 33. | Syed IU. Feminist Political Economy of Health: Current Perspectives and Future Directions. Healthcare (Basel). 2021 Feb 22;9(2):233. doi: 10.3390/healthcare9020233. PMID: 33671484; PMCID: PMC7926791. | Review Article |
| 34. | Meagher K, Attal B, Patel P. Exploring the role of gender and women in the political economy of health in armed conflict: a narrative review. Global Health. 2021 Aug 4;17(1):88. doi: 10.1186/s12992-021-00738-9. PMID: 34348740; PMCID: PMC8334332. | Review article |
| 35. | Walls H, Nisbett N, Laar A, Drimie S, Zaidi S, Harris J. Addressing Malnutrition: The Importance of Political Economy Analysis of Power. Int J Health Policy Manag. 2021 Dec 1;10(12):809-816. doi: 10.34172/ijhpm.2020.250. PMID: 33590738; PMCID: PMC9309968. | Review article |
| 36. | Swami S, Srivastava T. Role of Culture, Values, and Politics in the Implementation of Health Technology Assessment in India: A Commentary. Value Health. 2020 Jan;23(1):39-42. doi: 10.1016/j.jval.2019.10.002. Epub 2019 Dec 6. PMID: 31952672. | Commentary |
| 37. | Dada S, Ashworth HC, Bewa MJ, Dhatt R. Words matter: political and gender analysis of speeches made by heads of government during the COVID-19 pandemic. BMJ Glob Health. 2021 Jan;6(1):e003910. doi: 10.1136/bmjgh-2020-003910. PMID: 33514593; PMCID: PMC7849321. | No structured political economy analysis |
| 38. | Turk F. Data generalizability, data transferability, and the political economy of pharmacoeconomic guidelines. Value Health. 2010 Dec;13(8):863-4. doi: 10.1111/j.1524-4733.2010.00766.x. PMID: 20659271. | Editorial |
| 39. | Campbell M, Escobar O, Fenton C, Craig P. The impact of participatory budgeting on health and wellbeing: a scoping review of evaluations. BMC Public Health. 2018 Jul 3;18(1):822. doi: 10.1186/s12889-018-5735-8. PMID: 29970044; PMCID: PMC6029380. | Review Article |
| 40. | Hinck P, Gutierrez-Colosía M, Duval C, König HH, Simon J, Fischer C, Mayer S, Salvador-Carulla L, Brodszky V, Roijen LH, Evers S, Park AL, Hollingworth W; PECUNIA Group; Konnopka A. The identification of economically relevant health and social care services for mental disorders in the PECUNIA project. BMC Health Serv Res. 2023 Sep 29;23(1):1045. doi: 10.1186/s12913-023-09944-0. PMID: 37775752; PMCID: PMC10542258. | No structured political economy analysis |
| 41. | Gilson L, Shroff ZC, Shung-King M. Introduction to the Special Issue on "Analysing the Politics of Health Policy Change in Low- and Middle-Income Countries: The HPA Fellowship Programme 2017-2019". Int J Health Policy Manag. 2021 Jul 1;10(7):360-363. doi: 10.34172/ijhpm.2021.43. PMID: 33949819; PMCID: PMC9056138. | Editorial |
| 42. | Sedighi T, Varga L, Hosseinian-Far A, Daneshkhah A. Economic Evaluation of Mental Health Effects of Flooding Using Bayesian Networks. Int J Environ Res Public Health. 2021 Jul 13;18(14):7467. doi: 10.3390/ijerph18147467. PMID: 34299916; PMCID: PMC8303130. | No structured political economy analysis |
| 43. | Khanal GN, Bharadwaj B, Upadhyay N, Bhattarai T, Dahal M, Khatri RB. Evaluation of the National Health Insurance Program of Nepal: are political promises translated into actions? Health Res Policy Syst. 2023 Jan 20;21(1):7. doi: 10.1186/s12961-022-00952-w. PMID: 36670433; PMCID: PMC9862822. | Review article |
| 44. | Shretta R, Avanceña AL, Hatefi A. The economics of malaria control and elimination: a systematic review. Malar J. 2016 Dec 12;15(1):593. doi: 10.1186/s12936-016-1635-5. PMID: 27955665; PMCID: PMC5154116. | Review article |
| 45. | Walt G, Gilson L. Reforming the health sector in developing countries: the central role of policy analysis. Health policy and planning. 1994 Dec 1;9(4):353-70. | Review Article |
| 46. | Etiaba E, Uguru N, Ebenso B, Russo G, Ezumah N, Uzochukwu B, Onwujekwe O. Development of oral health policy in Nigeria: an analysis of the role of context, actors and policy process. BMC Oral Health. 2015 May 6;15:56. doi: 10.1186/s12903-015-0040-8. PMID: 25943102; PMCID: PMC4424590. | Review article |
| 47. | Walt G, Shiffman J, Schneider H, Murray SF, Brugha R, Gilson L. ‘Doing’health policy analysis: methodological and conceptual reflections and challenges. Health policy and planning. 2008 Sep 1;23(5):308-17. | Review article |
| 48. | Chen S, Chen Y, Feng Z, Chen X, Wang Z, Zhu J, Jin J, Yao Q, Xiang L, Yao L, Sun J, Zhao L, Fung H, Wong EL, Dong D. Barriers of effective health insurance coverage for rural-to-urban migrant workers in China: a systematic review and policy gap analysis. BMC Public Health. 2020 Mar 30;20(1):408. doi: 10.1186/s12889-020-8448-8. PMID: 32228665; PMCID: PMC7106835. | Review article |
| 49. | Morgan-Trimmer S. Policy is political; our ideas about knowledge translation must be too. J Epidemiol Community Health. 2014 Nov 1;68(11):1010-1. | Editorial |
| 50. | Witter S, Boukhalfa C, Cresswell JA, Daou Z, Filippi V, Ganaba R, Goufodji S, Lange IL, Marchal B, Richard F, FEMHealth team. Cost and impact of policies to remove and reduce fees for obstetric care in Benin, Burkina Faso, Mali and Morocco. International journal for equity in health. 2016 Dec;15:1-9. | No structured political economy analysis |
| 51. | Rizvi SS, Douglas R, Williams OD, Hill PS. The political economy of universal health coverage: a systematic narrative review. Health policy and planning. 2020 Apr;35(3):364-72. | Review article |
| 52. | Tumwine JK. Putting back basic sciences, political economy and sexuality into health. Afr Health Sci. 2011 Dec;11(4):524-5. PMID: 22649430; PMCID: PMC3362979. | Editorial |
| 53. | Saleh S, Fouad FM. Political economy of health in fragile and conflict-affected regions in the Middle East and North Africa region. J Glob Health. 2022 Aug 13;12:01003. doi: 10.7189/jogh.12.01003. PMID: 35959965; PMCID: PMC9373566. | Editorial |
| 54. | Jakovljevic M, Liu Y, Cerda A, Simonyan M, Correia T, Mariita RM, Kumara AS, Garcia L, Krstic K, Osabohien R, Toan TK, Adhikari C, Chuc NTK, Khatri RB, Chattu VK, Wang L, Wijeratne T, Kouassi E, Khan HN, Varjacic M. The Global South political economy of health financing and spending landscape - history and presence. J Med Econ. 2021 Nov;24(sup1):25-33. doi: 10.1080/13696998.2021.2007691. PMID: 34866543. | Review article |
| 55. | Baker P, Lacy-Nichols J, Williams O, Labonté R. The Political Economy of Healthy and Sustainable Food Systems: An Introduction to a Special Issue. Int J Health Policy Manag. 2021 Nov 1;10(12):734-744. doi: 10.34172/ijhpm.2021.156. PMID: 34836454; PMCID: PMC9309973. | Editorial |
| 56. | Scott-Marshall HK. A Political Economy Lens on Health Inequalities in the 21st Century. Am J Public Health. 2021 Apr;111(4):553-555. doi: 10.2105/AJPH.2021.306161. PMID: 33689434; PMCID: PMC7958037. | Editorial |
| 57. | Kittelsen SK, Fukuda-Parr S, Storeng KT. The political determinants of health inequities and universal health coverage. Globalization and Health. 2019 Nov;15:1-5. | Editorial |
| 58. | Shiffman J. Political context and health financing reform. Health Systems & Reform. 2019 Jul 3;5(3):257-9. | Commentary |
| 59. | Roy V, Hamilton D, Chokshi DA. Health and political economy: building a new common sense in the United States. Health Aff Sch. 2024 May 6;2(5):qxae041. doi: 10.1093/haschl/qxae041. PMID: 38757005; PMCID: PMC11071682. | No structured political economy analysis |
| 60. | Sott MK, Bender MS, da Silva Baum K. Covid-19 Outbreak in Brazil: Health, Social, Political, and Economic Implications. Int J Health Serv. 2022 Oct;52(4):442-454. doi: 10.1177/00207314221122658. Epub 2022 Sep 4. PMID: 36062608; PMCID: PMC9445630. | Review Article |
| 61. | Mecaskey J, Verboom B, Liverani M, Mijumbi-Deve R, Jessani NS. Improving institutional platforms for evidence-informed decision-making: getting beyond technical solutions. Health Res Policy Syst. 2023 Jan 16;21(1):5. doi: 10.1186/s12961-022-00948-6. PMID: 36647051; PMCID: PMC9841961. | Editorial |
| 62. | Coelho GLH, Hanel PHP, Vilar R, Monteiro RP, Cardoso FJV, Gouveia VV. Who prioritizes the economy over health? The role of political orientation and human values. Pers Individ Dif. 2021 Sep;179:110890. doi: 10.1016/j.paid.2021.110890. Epub 2021 Apr 16. PMID: 34866722; PMCID: PMC8631548. | No structured political economy analysis |
| 63. | Reddy SK, Mazhar S, Lencucha R. The financial sustainability of the World Health Organization and the political economy of global health governance: a review of funding proposals. Global Health. 2018 Nov 29;14(1):119. doi: 10.1186/s12992-018-0436-8. PMID: 30486890; PMCID: PMC6264055. | Review article |
| 64. | Tangcharoensathien V, Panichkriangkrai W, Witthyapipopsakul W, Patcharanarumol W. COVID-19 Aftermath: Direction Towards Universal Health Coverage in Low-Income Countries Comment on "Health Coverage and Financial Protection in Uganda: A Political Economy Perspective". Int J Health Policy Manag. 2023;12:7519. doi: 10.34172/ijhpm.2022.7519. Epub 2022 Sep 14. PMID: 36243945; PMCID: PMC10125230. | Commentary |
| 65. | Boxall AM, Short SD. Political economy and population health: is Australia exceptional? Aust New Zealand Health Policy. 2006 Jun 1;3:6. doi: 10.1186/1743-8462-3-6. PMID: 16737549; PMCID: PMC1513585. | Commentary |
| 66. | Lowe M. Obesity and climate change mitigation in Australia: overview and analysis of policies with co-benefits. Aust N Z J Public Health. 2014 Feb;38(1):19-24. doi: 10.1111/1753-6405.12150. PMID: 24494940. | Review article |
| 67. | Kim CB. A Historical Legacy for Universal Health Coverage in the Republic of Korea: Moving Towards Health Coverage and Financial Protection in Uganda Comment on "Health Coverage and Financial Protection in Uganda: A Political Economy Perspective". Int J Health Policy Manag. 2023;12:7434. doi: 10.34172/ijhpm.2023.7434. Epub 2023 Feb 13. PMID: 37579462; PMCID: PMC10125087. | Commentary |
| 68. | Sathyamala C. COVID-19: The Political Economy of a Global Pandemic. Dev Change. 2022 May 5:10.1111/dech.12711. doi: 10.1111/dech.12711. Epub ahead of print. PMID: 35942175; PMCID: PMC9348391. | Review article |
| 69. | Fox AM. Advancing Empirics and Theory for a Deeper Political Economy Analysis Comment on "Health Coverage and Financial Protection in Uganda: A Political Economy Perspective". Int J Health Policy Manag. 2023;12:7537. doi: 10.34172/ijhpm.2023.7537. Epub 2023 Sep 10. PMID: 38618806; PMCID: PMC10590232. | Commentary |
| 70. | Labonté R. From Mid-Level Policy Analysis to Macro-Level Political Economy Comment on "Developing a Framework for a Program Theory-Based Approach to Evaluating Policy Processes and Outcomes: Health in All Policies in South Australia". Int J Health Policy Manag. 2018 Jul 1;7(7):656-658. doi: 10.15171/ijhpm.2018.12. PMID: 29996585; PMCID: PMC6037494. | Commentary |
| 71. | Macintyre AK, Shipton D, Sarica S, Scobie G, Craig N, McCartney G. Assessing the effects of population-level political, economic and social exposures, interventions and policies on inclusive economy outcomes for health equity in high-income countries: a systematic review of reviews. Syst Rev. 2024 Feb 8;13(1):58. doi: 10.1186/s13643-023-02429-5. PMID: 38331910; PMCID: PMC10851517. | Review article |
| 72. | Martínez-Córdoba PJ, Benito B, García-Sánchez IM. Efficiency in the governance of the Covid-19 pandemic: political and territorial factors. Global Health. 2021 Sep 21;17(1):113. doi: 10.1186/s12992-021-00759-4. PMID: 34548073; PMCID: PMC8454294. | Review article |
| 73. | Adamu AA, Essoh TA, Jalo RI, Wiysonge CS. Toward political economy of sustainable financing for immunization in the World Health Organization African Region through a systems thinking lens. Int J Infect Dis. 2023 Nov;136:158-161. doi: 10.1016/j.ijid.2023.09.017. Epub 2023 Sep 27. PMID: 37774773. | No structured political economy analysis |
| 74. | Muntaner C, Borrell C, Ng E, Chung H, Espelt A, Rodriguez-Sanz M, Benach J, O'Campo P. Politics, welfare regimes, and population health: controversies and evidence. Sociol Health Illn. 2011 Sep;33(6):946-64. doi: 10.1111/j.1467-9566.2011.01339.x. PMID: 21899562. | Review article |
| 75. | Kalateh Sadati A, Iman MT, Bagheri Lankarani K. Medical paraclinical standards, political economy of clinic, and patients' clinical dependency; a critical conversation analysis of clinical counseling in South of iran. Int J Community Based Nurs Midwifery. 2014 Jul;2(3):157-68. PMID: 25349858; PMCID: PMC4201202. | No structured political economy analysis |
| 76. | Cotula L. Towards a political economy of the COVID-19 crisis: Reflections on an agenda for research and action. World Dev. 2021 Feb;138:105235. doi: 10.1016/j.worlddev.2020.105235. Epub 2020 Oct 16. PMID: 33100479; PMCID: PMC7566792. | Commentary |
| 77. | Obilade TT. The Political Economy of the Ebola Virus Disease (EVD); Taking Individual and Community Ownership in the Prevention and Control of EVD. Healthcare (Basel). 2015 Jan 28;3(1):36-49. doi: 10.3390/healthcare3010036. PMID: 27417746; PMCID: PMC4934522. | Review article |
| 78. | McMurray A. Empowerment and enterprise: the political economy of nursing. Collegian. 2010;17(3):113-8. doi: 10.1016/j.colegn.2009.12.001. PMID: 21046964. | No structured political economy analysis |
| 79. | Loffreda G, Arakelyan S, Bou-Orm I, Holmer H, Allen LN, Witter S, Ager A, Diaconu K. Barriers and Opportunities for WHO "Best Buys" Non-communicable Disease Policy Adoption and Implementation From a Political Economy Perspective: A Complexity Systematic Review. Int J Health Policy Manag. 2024;13:7989. doi: 10.34172/ijhpm.2023.7989. Epub 2024 Feb 4. PMID: 38618832; PMCID: PMC11016278. | Review article |
| 80. | Barlow P. COVID-19, Trade, and Health: This Changes Everything? Comment on "What Generates Attention to Health in Trade Policy-Making? Lessons From Success in Tobacco Control and Access to Medicines: A Qualitative Study of Australia and the (Comprehensive and Progressive) Trans-Pacific Partnership". Int J Health Policy Manag. 2022 Apr 1;11(4):525-528. doi: 10.34172/ijhpm.2020.220. PMID: 33233035; PMCID: PMC9309943. | Commentary |
| 81. | Stewart J. The political economy of the British National Health Service, 1945-1975: opportunities and constraints? Med Hist. 2008 Oct;52(4):453-70. doi: 10.1017/s0025727300000181. PMID: 18958249; PMCID: PMC2570443. | Commentary |
| 82. | Schram A, Goldman S. Paradigm Shift: New Ideas for a Structural Approach to NCD Prevention Comment on "How Neoliberalism Is Shaping the Supply of Unhealthy Commodities and What This Means for NCD Prevention". Int J Health Policy Manag. 2020 Mar 1;9(3):124-127. doi: 10.15171/ijhpm.2019.105. PMID: 32202097; PMCID: PMC7093042. | Commentary |
| 83. | Fox DM. Comment: epidemiology and the new political economy of medicine. Am J Public Health. 1999 Apr;89(4):493-6. doi: 10.2105/ajph.89.4.493. PMID: 10191789; PMCID: PMC1508906. | Commentary |
| 84. | Renzaho AMN. The Need for the Right Socio-Economic and Cultural Fit in the COVID-19 Response in Sub-Saharan Africa: Examining Demographic, Economic Political, Health, and Socio-Cultural Differentials in COVID-19 Morbidity and Mortality. Int J Environ Res Public Health. 2020 May 15;17(10):3445. doi: 10.3390/ijerph17103445. PMID: 32429123; PMCID: PMC7277405. | No structured political economy analysis |
| 85. | Collins C, Green A, Hunter D. Health sector reform and the interpretation of policy context. Health policy. 1999 Apr 1;47(1):69-83. | Commentary |
| 86. | Peabody JW. Economic reform and health sector policy: lessons from structural adjustment programs. Social science & medicine. 1996 Sep 1;43(5):823-35. | No structured political economy analysis |
| 87. | Standing H. An overview of changing agendas in health sector reforms. Reproductive health matters. 2002 Nov 1;10(20):19-28. | Commentary |
| 88. | Callen M, Gulzar S, Hasanain SA, Khan MY. The political economy of public sector absence: experimental evidence from Pakistan. National Bureau of Economic Research; 2016 Jun 13. | No structured political economy analysis |
| 89. | Berman P, Bossert T. A decade of health sector reform in developing countries: what have we learned. Washington, UNAID. 2000 Mar 15. | Commentary |
| 90. | Cassels A. Health sector reform: key issues in less developed countries. Journal of International development. 1995 May;7(3):329-47. | Review article |
| 91. | Connelly J. A realistic theory of health sector management: the case for critical realism. Journal of management in medicine. 2000 Dec 1;14(5/6):262-71. | Concept paper |
| 92. | Mahmood Q, Muntaner C. Politics, class actors, and health sector reform in Brazil and Venezuela. Global Health Promotion. 2013 Mar;20(1):59-67. | No structured political economy |
| 93. | Tsofa BK. Examining the effects of political decentralisation in Kenya on health sector planning and budgeting: a case study of Kilifi County (Doctoral dissertation, London School of Hygiene & Tropical Medicine). | No structured political economy |
| 94. | Asquith A, Brunton M, Robinson D. Political influence on public–private partnerships in the public health sector in New Zealand. International Journal of Public Administration. 2015 Feb 23;38(3):179-88. | No structured political economy |
| 95. | Sell SK, Williams OD. Health under capitalism: a global political economy of structural pathogenesis. Review of International Political Economy. 2020 Jan 2;27(1):1-25. | Commentary |
| 96. | Ogeya M, Lambe F. The political economy of mini-grid electricity development and innovation in Kenya. Renewable and Sustainable Energy Transition. 2025 Feb 1;6:100092. | Not related to health |
| 97. | Waitzberg R, Pfundstein I, Maresso A, Rechel B, van Ginneken E, Quentin W. Health system description and assessment: a scoping review of templates for systematic analyses. Health Research Policy and Systems. 2024 Jul 11;22(1):82. | Review article |
| 98. | Paina L, Young R, Oladapo O, Leandro J, Chen Z, Igusa T. Prospective policy analysis—a critical interpretive synthesis review. Health policy and planning. 2024 May 1;39(4):429-41. | Review article |
| 99. | Kulkarni S, Shukla A. Poor sanitation, water scarcity, and the political economy of sugarcane in Maharashtra, India. Bernadette P. Resurrección, Professor, Global Development Studies, Queen’s University, Canada. 2025:253. | Not related to health |
| 100. | Karuveettil V, Janakiram C, John D, Mathur M, Varma B, Green H. Political economy analysis of health: a scoping review protocol. JBI Evidence Synthesis. 2024 Sep 1;22(9):1906-13. | Study protocol |
| 101. | Amboko B, Nzinga J, Tsofa B, Mugo P, Musiega A, Maritim B, Wong E, Mazzilli C, Ng’ang’a W, Hagedorn B, Turner G. Evaluating the impact, implementation experience and political economy of primary care networks in Kenya: protocol for a mixed methods study. Health Research Policy and Systems. 2025 Jan 27;23(1):14. | Study protocol |
| 102. | Sengupta, Shruti and Kumar, Deepak and Yadav, Sudha, Does Women's Political Inclusion Reduce Gender Bias in Health Outcomes? Evidence from India (October 24, 2024). Available at SSRN: https://ssrn.com/abstract=5123953 | Only the political aspect was covered; economic analysis was lacking. |
| 103. | Heller JC, Givens ML, Johnson SP, Kindig DA. Keeping it political and powerful: defining the structural determinants of health. Milbank Q. 2024; 102(2): 351-366. https://doi.org/10.1111/1468-0009.12695 | Only the political aspect was covered; economic analysis was lacking. |
| 104. | Loffreda, G., Arakelyan, S., Bou-Orm, I., Holmer, H., Allen, L. N., Witter, S., Ager, A., Diaconu, K. Barriers and Opportunities for WHO ‘Best Buys’ Non-Communicable Disease Policy Adoption and Implementation From a Political Economy Perspective: A Complexity Systematic Review. International Journal of Health Policy and Management, 2023; 12(Issue 1): 1-14. doi: 10.34172/ijhpm.2023.7989 | Only the political aspect was covered; economic analysis was lacking. |
